# Deep learning-based anomaly detection for precision field crop protection

**DOI:** 10.3389/fpls.2025.1576756

**Published:** 2025-05-14

**Authors:** Cheng Wei, Yifeng Shan, MengZhe Zhen

**Affiliations:** ^1^ Chongqing Business Vocational College, Chongqing, China; ^2^ Ningbo University of Finance and Economics, School of Basic Education, Ningbo, Zhejiang, China; ^3^ Ningbo University of Finance and Economics, School of Digital Technology and Engineering, Ningbo, Zhejiang, China

**Keywords:** precision agriculture, anomaly detection, multi-modal data fusion, resource optimization, sustainable farming

## Abstract

**Introduction:**

Precision agriculture relies on advanced technologies to optimize crop protection and resource utilization, ensuring sustainable and efficient farming practices. Anomaly detection plays a critical role in identifying and addressing irregularities, such as pest outbreaks, disease spread, or nutrient deficiencies, that can negatively impact yield. Traditional methods struggle with the complexity and variability of agricultural data collected from diverse sources.

**Methods:**

To address these challenges, we propose a novel framework that integrates the Integrated Multi-Modal Smart Farming Network (IMSFNet) with the Adaptive Resource Optimization Strategy (AROS). IMSFNet employs multimodal data fusion and spatiotemporal modeling to provide accurate predictions of crop health and yield anomalies by leveraging data from UAVs, satellites, ground sensors, and weather stations. AROS dynamically optimizes resource allocation based on real-time environmental feedback and multi-objective optimization, balancing yield maximization, cost efficiency, and environmental sustainability.

**Results:**

Experimental evaluations demonstrate the effectiveness of our approach in detecting anomalies and improving decision-making in precision agriculture.

**Discussion:**

This framework sets a new standard for sustainable and data-driven crop protection strategies.

## 1 Introduction

Precision agriculture has revolutionized the agricultural industry, enabling efficient and sustainable crop management through targeted interventions. Within this domain, anomaly detection plays a critical role in field crop protection by identifying early signs of diseases, pests, nutrient deficiencies, or other stress factors that compromise crop health Zhang et al. (2024b). Not only does early detection help reduce the overuse of chemical inputs such as pesticides and fertilizers, but it also minimizes yield losses and supports sustainable farming practices Gao et al. (2024a). Traditional anomaly detection techniques, while effective in controlled conditions, often fail to capture the complexity of real-world agricultural systems, which involve heterogeneous data sources, dynamic environmental conditions, and intricate interactions between crops and external stressors Zhu et al. (2024a). As a result, deep learning-based methods have emerged as a promising solution for improving the accuracy and scalability of anomaly detection in precision field crop protection. By leveraging multi-modal data from satellite imagery, drones, and on-ground sensors, these methods provide an end-to-end framework for identifying and addressing crop health anomalies Li et al. (2021).

Early approaches to anomaly detection in agriculture were based on rule-based systems and statistical models, which relied on domain knowledge and handcrafted features to identify deviations from normal conditions Zavrtanik et al. (2021). For example, threshold-based methods used predefined values for parameters like vegetation indices or soil moisture levels to detect anomalies Deng and Hooi (2021). Similarly, statistical models such as principal component analysis (PCA) and clustering were used to identify outliers in crop health data Zou et al. (2022). While these methods provided interpretable results and were computationally efficient, they lacked the ability to generalize across diverse environmental conditions and crop types Bergmann et al. (2021). Furthermore, their reliance on fixed thresholds and handcrafted features made them inadequate for capturing the complex, non-linear patterns associated with crop health anomalies caused by diseases or pests Gudovskiy et al. (2021).

The shift toward data-driven approaches introduced machine learning algorithms capable of learning patterns from historical data to improve anomaly detection You et al. (2022). Techniques such as support vector machines (SVMs), random forests, and k-nearest neighbors were employed to classify crop health statuses based on features extracted from remote sensing or field data Liu et al. (2023a). These models achieved better adaptability compared to rule-based systems, as they could learn relationships between input features and anomalies without explicit thresholds. For example, machine learning models were applied to identify plant stress from hyperspectral imagery or to classify pest infestations based on soil and weather data Zhu et al. (2024b). However, these approaches faced limitations in their ability to handle highdimensional and multi-modal data, such as the integration of spectral, spatial, and temporal information. Traditional machine learning methods required extensive feature engineering and struggled to generalize to new datasets or unseen conditions Tian et al. (2021).

Deep learning has transformed anomaly detection in precision agriculture by introducing architectures that can automatically learn hierarchical representations of crop health data Han et al. (2022). Convolutional neural networks (CNNs) have been widely used for analyzing spatial patterns in satellite and drone imagery, enabling the detection of disease outbreaks, pest infestations, and nutrient deficiencies. Recurrent neural networks (RNNs) and long short-term memory (LSTM) networks have been employed to model temporal changes in crop health, such as monitoring vegetation growth over a growing season Jiang et al. (2023). Furthermore, generative adversarial networks (GANs) and autoencoders have demonstrated success in unsupervised anomaly detection, where models are trained on normal data to identify deviations that signify anomalies Zhang et al. (2024c). For example, autoencoders have been used to detect stress signals in hyperspectral images by reconstructing healthy crop patterns and flagging deviations as anomalies Tien et al. (2023). Despite these advancements, challenges remain, particularly in the integration of multi-modal data from diverse sources, the interpretability of deep learning models, and the scalability of these methods for large-scale agricultural applications Wyatt et al. (2022).

Recent works in spatiotemporal modeling for agricultural anomaly detection primarily rely on CNNs and LSTMs to process spatial and sequential data, respectively. For example, proposed a hybrid CNN-LSTM model Xu et al. (2021) to capture vegetation dynamics from satellite images, but their approach struggled with long-range dependencies and multimodal data integration. Similarly, introduced an attention-enhanced LSTM Tuli et al. (2022) for pest detection, yet the model’s performance degraded with increasing data heterogeneity. In contrast, our proposed IMSFNet incorporates GNNs to model spatial relationships between crop regions, while employing Transformers to capture long-term temporal dependencies more effectively than LSTMs. This hybrid architecture allows for adaptive weighting of multimodal information, improving the robustness and interpretability of anomaly detection in precision agriculture.

To address these challenges, we propose a novel deep learning-based framework for anomaly detection tailored for precision field crop protection. The proposed framework integrates multi-modal data from satellite imagery, drones, and *in-situ* sensors to capture a holistic view of crop health. A hybrid architecture combining convolutional neural networks and graph neural networks (GNNs) is employed to model spatial dependencies between crops, while transformers are utilized to capture temporal patterns in crop growth and environmental conditions. The framework incorporates unsupervised learning techniques, such as variational autoencoders, to detect subtle anomalies that may not be present in labeled datasets. By prioritizing scalability and interpretability, this framework is designed to support real-time decision-making and adaptive interventions, ultimately enhancing crop resilience and productivity.

We summarize our contributions as follows:

- We propose IMSFNet, a novel Integrated Multi-Modal Smart Farming Network, which combines GNNs for spatial dependency modeling and Transformers for long-term temporal feature extraction. This hybrid architecture effectively captures crop health variations and environmental anomalies across different spatial and temporal scales.
- We introduce a multi-modal fusion strategy that integrates satellite imagery, UAV-based imaging, ground sensors, and meteorological data. Unlike previous works that rely on independent processing pipelines, IMSFNet jointly learns representations from heterogeneous data sources, leading to improved anomaly detection performance.
- We develop AROS (Adaptive Resource Optimization Strategy), a real-time optimization framework that dynamically adjusts resource allocation based on multi-objective optimization. AROS leverages reinforcement learning-based feedback mechanisms to improve efficiency and sustainability in precision agriculture.
- Our extensive experiments on multiple real-world datasets demonstrate that IMSFNet and AROS achieve 3.12% higher F1-score and 2.89% higher accuracy compared to state-of-the-art anomaly detection models. We release our implementation and dataset annotations to facilitate further research in deep learning-based precision agriculture.

## 2 Related work

### 2.1 Deep learning for anomaly detection in agriculture

Anomaly detection is a critical component of precision agriculture, aiming to identify abnormal patterns in crop health, pest infestations, and environmental conditions Wang et al. (2023a). Traditional anomaly detection methods, such as threshold-based techniques and classical machine learning models like Support Vector Machines (SVMs) and k-Nearest Neighbors (k-NN), often fail to capture the complexity of agricultural environments Wang et al. (2023b). Deep learning approaches have emerged as more robust alternatives, leveraging their ability to process large-scale, high-dimensional data and uncover complex, nonlinear relationships Defard et al. (2020). In agricultural applications, CNNs are widely employed to analyze visual data, such as images from drones and ground-based cameras Gao et al. (2024b). For instance, CNN-based models can detect visual anomalies in crops, such as discoloration, irregular growth, and pest damage Park et al. (2020). RNNs and LSTM networks, on the other hand, are applied to sequential data, such as time-series environmental sensor readings, to identify trends or deviations indicative of anomalies DeMedeiros et al. (2023). Recent advancements include hybrid models that integrate CNNs and RNNs to analyze spatiotemporal data, such as videos of crop fields over time Batzner et al. (2023). These models enable the detection of anomalies not just at a single point in time but also in evolving patterns, such as the spread of a disease across a field Feng et al. (2021). While these methods show promise, challenges remain in dealing with noisy and imbalanced data, where anomalies constitute only a small fraction of the dataset. Techniques such as data augmentation, synthetic anomaly generation, and adversarial training are being developed to address these issues and enhance model robustness in real-world scenarios Audibert et al. (2020).

### 2.2 Multi-modal data fusion for precision crop protection

Precision crop protection often requires integrating data from multiple sources, such as remote sensing, IoT devices, and weather stations, to effectively detect and respond to anomalies Le and Zhang (2021). Multi-modal data fusion enables the combination of these diverse data types to improve the accuracy and reliability of anomaly detection systems Liu et al. (2021). Deep learning has been instrumental in facilitating such fusion, with models that can process and integrate heterogeneous data modalities. For visual data, drone and satellite imagery are commonly used to monitor crop health, while IoT devices provide real-time sensor readings for soil moisture, temperature, and humidity Salehi et al. (2020). Multi-modal architectures, such as those combining CNNs for image analysis with fully connected or Transformer layers for sensor data, have demonstrated improved performance in identifying anomalies such as nutrient deficiencies, water stress, and pest outbreaks Liu et al. (2023b). For example, attention mechanisms have been employed to prioritize the most relevant data sources for decision-making, enhancing the interpretability of these systems. Temporal fusion models, such as Temporal Fusion Transformers (TFTs), have also been applied to integrate time-series data from multiple sensors with historical climate records, enabling more accurate anomaly predictions Roth et al. (2021). GNNs are another emerging approach for multi-modal fusion, particularly in representing spatial relationships within a field, such as proximity between affected regions or the spread of an anomaly across neighboring plots. Despite the benefits, multi-modal fusion faces challenges such as data heterogeneity, missing values, and high computational requirements. Advances in self-supervised learning and imputation techniques are being explored to address these limitations, enabling models to learn meaningful representations from incomplete or noisy datasets Deng and Li (2022). Furthermore, edge computing and hardware acceleration are being investigated to enable real-time data fusion and anomaly detection in resource-constrained agricultural environments.

### 2.3 Applications of anomaly detection in crop protection

Deep learning-based anomaly detection has found numerous applications in precision crop protection, addressing challenges such as pest infestations, disease outbreaks, and abiotic stress factors like drought and frost Ni et al. (2018). By automating the identification of anomalies, these systems reduce the reliance on manual scouting, which is labor-intensive and prone to errors, especially in large-scale agricultural settings Ni et al. (2017). For pest detection, models leveraging CNNs and object detection frameworks like YOLO (You Only Look Once) have been used to identify specific pest species in field images. These models enable targeted interventions, such as pesticide application, reducing chemical usage and minimizing environmental impact Mishra et al. (2021). Similarly, for disease detection, segmentation models such as U-Net and Mask R-CNN have been employed to localize affected areas, allowing for precise treatment. Beyond visual data, deep learning models have been applied to sensor-based anomaly detection. For example, LSTM networks are used to analyze soil moisture and temperature data to identify water stress, while Variational Autoencoders (VAEs) Ni et al. (2016) and GANs are employed to detect deviations from normal patterns in multi-dimensional sensor data. These approaches are particularly effective in identifying early warning signs of crop stress, enabling timely interventions that mitigate yield losses. Another application lies in predicting the spatial spread of anomalies, such as the propagation of pests or diseases within a field. Spatiotemporal models, including 3D CNNs and ST-GCNs (Spatio-Temporal Graph Convolutional Networks), are used to predict how anomalies evolve over time and space, aiding in the design of containment strategies. For instance, these models can simulate the effects of varying weather conditions on the spread of an anomaly, providing actionable insights for farmers and agronomists. While these applications demonstrate the potential of anomaly detection in crop protection, practical deployment remains challenging due to factors such as limited labeled data, variability in agricultural environments, and the need for domain-specific customization. Future research should focus on developing scalable and generalizable models, incorporating domain knowledge into deep learning frameworks, and ensuring the ethical use of these technologies in precision agriculture.

## 3 Method

### 3.1 Overview

Precision agriculture, also referred to as smart farming or digital farming, is an advanced approach to agricultural management that leverages modern technologies such as remote sensing, geographic information systems (GIS), Internet of Things (IoT), and artificial intelligence (AI) to optimize crop production and resource utilization. The goal of precision agriculture is to enhance efficiency, reduce environmental impact, and increase yield through the precise monitoring and management of agricultural inputs, including water, fertilizers, and pesticides. Traditional farming practices often involve uniform treatment of large agricultural fields, which can lead to inefficiencies and overuse of resources. By contrast, precision agriculture adopts a data-driven approach, tailoring interventions to the specific needs of crops, soil conditions, and environmental factors. This is achieved through a combination of advanced technologies that collect, analyze, and act upon data at a granular level. For instance, sensors embedded in the soil can measure moisture and nutrient levels, drones equipped with multispectral cameras can capture crop health data, and machine learning algorithms can predict optimal planting and harvesting times. The implementation of precision agriculture can be broadly categorized into three main components: data collection, data analysis, and decision-making. Data collection involves the use of various sensing technologies, such as satellite imagery, UAVs (unmanned aerial vehicles), and IoT-enabled sensors, to capture real-time information about crops and the environment. This data is then analyzed using advanced computational techniques, including machine learning and statistical modeling, to derive actionable insights. The insights are used to make informed decisions, such as variable-rate application of fertilizers or automated irrigation scheduling. Despite its promising potential, the widespread adoption of precision agriculture faces several challenges, including the high cost of technology, lack of technical expertise among farmers, and limited access to high-speed internet in rural areas. Addressing these barriers requires interdisciplinary efforts that integrate engineering, computer science, agronomy, and socioeconomics.

This paper introduces a novel framework for precision agriculture that integrates advanced sensing technologies with deep learning-based predictive models to enhance the scalability and robustness of smart farming systems. The proposed method focuses on multi-modal data fusion, combining visual, thermal, and spectral data from UAVs and ground-based sensors to improve the accuracy of crop health assessment and yield prediction. The framework also incorporates adaptive optimization strategies to address varying environmental and climatic conditions, ensuring its applicability across diverse agricultural contexts. The remainder of this paper is organized as follows. In Section 3.2, we formalize the precision agriculture problem, introducing the necessary mathematical foundations and notations. Section 3.3 introduces our proposed Integrated Multi-Modal Smart Farming Network (IMSFNet), a system that integrates advanced sensing and modeling techniques for efficient crop monitoring. In Section 3.4, we present the Adaptive Resource Optimization Strategy (AROS), a novel approach designed to optimize resource allocation through real-time predictions and environmental feedback.

To enhance readability, we briefly summarize the key technical abbreviations (In **Table 1**) used throughout the manuscript. IMSFNet refers to the proposed Integrated Multi-Modal Smart Farming Network, while AROS denotes the Adaptive Resource Optimization Strategy. Core components include MFE (Multi-Modal Feature Extraction), CBS (Convolution + Batch Normalization + SiLU), CSPBlock (Cross Stage Partial Block), and PConv (Parametric Convolution). FFCA-YOLO and L-FFCA-YOLO represent backbone architectures with cross-attentive and lightweight designs. Additional modules such as SCNN (Spatial Convolutional Neural Network), DMO (Dynamic Multi-Objective Optimization), PRA (Prioritized Resource Allocation), and RFM (Real-Time Feedback Mechanism) further support resource-efficient anomaly detection. Abbreviations like UAV (Unmanned Aerial Vehicle), NDVI (Normalized Difference Vegetation Index), GNN (Graph Neural Network), and LSTM (Long Short-Term Memory) are used to represent standard sensing and modeling technologies in precision agriculture.

**Table 1:** List of technical abbreviations used throughout the manuscript.

Abbreviation	Full Term
IMSFNet	Integrated Multi-Modal Smart Farming Network
AROS	Adaptive Resource Optimization Strategy
MFE	Multi-Modal Feature Extraction
CBS	Convolution + Batch Normalization + SiLU
CSPBlock	Cross Stage Partial Block
CSPFaster Block	Enhanced Cross Stage Partial Block (optimized for speed)
PConv	Parametric Convolution
FFCA-YOLO	Feature-Focused Cross-Attentive YOLO
L-FFCA-YOLO	Lightweight FFCA-YOLO
SCNN	Spatial Convolutional Neural Network
DMO	Dynamic Multi-Objective Optimization
PRA	Prioritized Resource Allocation
RFM	Real-Time Feedback Mechanism
NDVI	Normalized Difference Vegetation Index
IoT	Internet of Things
UAV	Unmanned Aerial Vehicle
RNN	Recurrent Neural Network
LSTM	Long Short-Term Memory
GAN	Generative Adversarial Network
VAE	Variational Autoencoder
GNN	Graph Neural Network
CNN	Convolutional Neural Network
TFT	Temporal Fusion Transformer
Q, K, V	Query, Key, Value (in attention mechanism)

### 3.2 Preliminaries

Precision agriculture relies on detecting anomalies in crop health to optimize resource allocation. Given an agricultural field *F* partitioned into *N* management zones $\left\{Z_{1},\;Z_{2}\ldots ,Z_{N}\right\}$ , each zone is represented by a feature vector *x_i_* derived from multi-modal data sources. The objective of anomaly detection is to identify zones where the observed feature vector *x_i_* deviates significantly from normal patterns. Let *p*(*x*) represent the probability distribution of normal crop health conditions. A zone *Z_i_* is classified as anomalous if its feature vector satisfies (Equation 1):


(1) $$\mathbb{P}(x_{i}|\theta )<\tau ,$$

where *θ* denotes the parameters of the learned normal distribution, and *τ* is a predefined anomaly threshold. To quantify anomalies, an anomaly score function $s_{i}$ is defined based on the deviation of *x_i_* from the mean feature vector *µ* of normal samples (Equation 2):


(2) $$s_{i}=D(x_{i},\mu ),$$

where *D*(·,·) represents a distance metric such as the Mahalanobis distance or Euclidean distance. Higher values of *s_i_* indicate stronger deviations from normal conditions, guiding adaptive interventions in precision agriculture.

The optimization objective can be formulated as (Equation 3):


(3) $$\underset{U}{\max}\sum_{i=1}^{N}Y_{i}(U_{i},p_{i})-\lambda C(U),$$

where $Y_{i}$ is the yield function for zone *Z_i_*, which depends on the intervention $U_{i}$ and the feature vector **p** *_i_*, and C(U) is the total cost function associated with the intervention strategy U . The parameter *λ* is a weighting factor that balances yield maximization and cost minimization.

Agricultural fields exhibit both spatial and temporal variability. Spatial variability arises from heterogeneities in soil properties, topography, and crop health across zones. Temporal variability is driven by dynamic environmental conditions, such as weather changes and seasonal cycles. Let **P**(*t*) = {**p**_1_(*t*), **p**_2_(*t*)*,…*, **p** *_N_* (*t*)} denote the time-dependent feature matrix for the field at time *t*. The evolution of crop yield $Y_{i}(t)$ for zone *Z_i_* can be modeled as (Equation 4):


(4) $$Y_{i}(t)=F(p_{i}(t),U_{i}(t),\eta_{i}(t)),$$

where F(·) is a function that encapsulates the complex relationships between environmental factors, interventions, and crop response, and $\eta_{i}(t)$ represents stochastic disturbances such as pests, diseases, or unpredicted weather events.

Precision agriculture integrates diverse types of multimodal data collected from various sensing technologies to effectively monitor crop and environmental conditions. Satellite imagery enables large-scale observation by providing vegetation indices, such as the Normalized Difference Vegetation Index (NDVI), and information about canopy cover. Unmanned aerial vehicles (UAVs), commonly referred to as drones, capture high-resolution visual, thermal, and multispectral images, offering detailed insights into crop health. Ground sensors contribute precise measurements of soil properties, including moisture, temperature, pH, and electrical conductivity, at specific locations within the field. Weather stations supply real-time data on environmental variables, including temperature, humidity, wind speed, and precipitation, further enhancing the decision-making process in precision agriculture.

Let $D=\{D_{\text{sat}},D_{\text{uav}},D_{\text{ground}},D_{\text{weather}}\}$ represent the collection of data from these sources. The fusion of multi-modal data is essential for developing a comprehensive understanding of field conditions.

In precision agriculture, decision-making often involves multiple competing objectives, such as maximizing yield, minimizing resource usage, and reducing environmental impact. The problem can be formulated as a multi-objective optimization (Equation 5):


(5) extmaximize [Y(U),−C(U),−E(U)],

where E(U) represents the environmental impact function. Multi-objective optimization techniques, such as Pareto front analysis or weighted sum methods, are employed to identify trade-offs and select optimal strategies.

### 3.3 Integrated multi-modal smart farming network

IMSFNet is an innovative deep learning framework designed for precision agriculture, seamlessly integrating multi-modal data sources to perform anomaly detection in a unified manner. Unlike conventional CNN-LSTM architectures, it leverages Graph Neural Networks to enhance spatial feature learning while incorporating Transformers to capture long-range temporal dependencies. This combination significantly improves the model’s ability to understand complex environmental interactions. The framework operates through a structured process that begins with extracting features from multiple data modalities, followed by an advanced fusion mechanism that integrates spatial and temporal information. It introduces a cross-modal interaction approach that strengthens the relationships between different data sources, ensuring a more comprehensive and accurate analysis of agricultural conditions.

**Figure 1:**
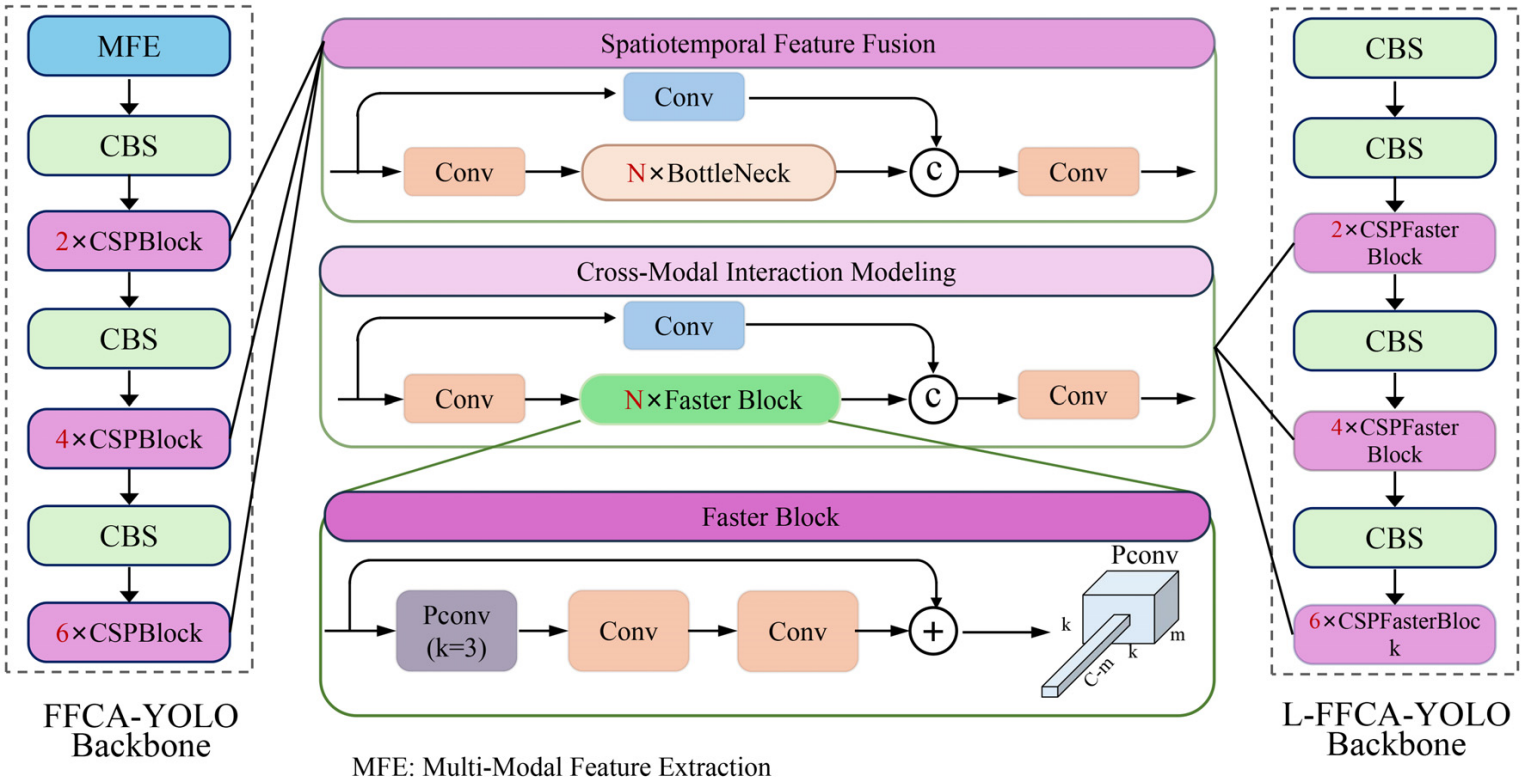
Overview of integrated multi-modal smart farming network architectures. The figure illustrates the structural components of the FFCA-YOLO and L-FFCA-YOLO backbones, highlighting key modules such as Multi-Modal Feature Extraction (MFE), Spatiotemporal Feature Fusion, Cross-Modal Interaction Modeling, and Faster Blocks. The FFCA-YOLO backbone consists of sequential CSPBlocks and CBS layers, while the L-FFCA-YOLO backbone incorporates CSPFaster Blocks for enhanced efficiency. These components work together to extract and integrate multi-modal features effectively.

IMSFNet integrates multiple data sources to achieve precise crop anomaly detection through multi-modal fusion, as illustrated in **Figure 1**. Each modality contributes a distinct feature set, including satellite imagery features represented as $X_{\text{sat}}\in {\mathbb{R}}^{H_{s}\times W_{s}\times d_{s}}$ , UAV imagery features as $X_{\text{uav}}\in {\mathbb{R}}^{H_{u}\times W_{u}\times d_{u}}$ , ground sensor features as $X_{\text{sens}}\in {\mathbb{R}}^{N_{g}\times d_{g}}$ , and weather data as $X_{\text{weather}}\in {\mathbb{R}}^{T_{w}\times d_{w}}$ . Each modality undergoes feature extraction through a modality-specific encoder *E_m_*, transforming the input into a latent representation *F_m_* = *E_m_* (*X_m_*) for all modalities, including satellite, UAV, sensor, and weather data. To ensure consistency across different modalities, all extracted features are projected into a common latent space ${\mathbb{R}}^{d}$ through modality-specific projection functions, expressed as *F*_proj_ *_,m_* = *P_m_* (*F_m_*). IMSFNet incorporates an attentionbased mechanism that dynamically assigns weights to each modality, enhancing the contribution of the most informative features. The final fused representation is computed as $F_{\text{fused}}=\sum_{m}w_{m}F_{m}$ , where the weights *w_m_* are determined using a softmax function applied to a learnable weight matrix *W_m_*, formulated as *w_m_* = softmax(*W_m_F_m_*). This fusion strategy enables IMSFNet to effectively integrate diverse agricultural data sources, leading to improved performance in crop anomaly detection.

#### 3.3.1 Multi-modal feature extraction

IMSFNet employs a robust multi-modal feature extraction process tailored to handle the diverse nature of input data sources, including satellite imagery, UAV-based imaging, ground sensors, and weather data. These data modalities provide complementary information critical for precision agriculture, capturing spatial, temporal, and environmental variability. Formally, let $D=\{D_{\text{sat}},D_{\text{uav}},D_{\text{ground}},D_{\text{weather}}\}$ denote the collection of input datasets. Each data source is independently processed through modalityspecific feature extractors $E_{\text{sat}},E_{\text{uav}},E_{\text{ground}},E_{\text{weather}}$ to generate low-dimensional feature embeddings **F**_sat_, **F**_uav_, **F**_ground_, **F**_weather_. The extraction process can be formalized as follows (Equations 6, 7):


(6) $$F_{\text{sat}}=E_{\text{sat}}(D_{\text{sat}}),\;F_{\text{uav}}=E_{\text{uav}}(D_{\text{uav}}),$$

(7) $$F_{\text{ground}}=E_{\text{ground}}(D_{\text{ground}}),\;F_{\text{weather}}=E_{\text{weather}}(D_{\text{weather}}).$$

Here, $E_{\text{sat}}$ processes satellite imagery to extract global spatial features, such as vegetation indices or canopy coverage, which are represented as $F_{\text{sat}}\in {\mathbb{R}}^{H_{s}\times W_{s}\times d_{s}}$ , where *H_s_*, *W_s_*, and *d_s_* correspond to the height, width, and feature dimensions of the satellite feature map. Similarly, $E_{\text{uav}}$ extracts high-resolution visual, thermal, and multispectral features from UAV-based imaging, producing $F_{\text{uav}}\in {\mathbb{R}}^{H_{u}\times W_{u}\times d_{u}}$ . Ground sensors, represented by $D_{\text{ground}}$ , provide point-level data on soil and environmental conditions, such as moisture, pH, and temperature. The extracted features $F_{\text{ground}}\in {\mathbb{R}}^{N_{g}\times d_{g}}$ capture the local variability across *N_g_* sensor locations. $E_{\text{weather}}$ processes meteorological data, such as temperature, humidity, and precipitation, into temporal embeddings $F_{\text{weather}}\in {\mathbb{R}}^{T_{w}\times d_{w}}$ , where *T_w_* denotes the time steps. To ensure consistency and facilitate downstream multi-modal fusion, each feature embedding is projected into a shared latent space of dimension *d* through a linear transformation $P_{m}$ specific to each modality *m* (Equation 8):


(8) $$F_{m}^{\text{proj}}=P_{m}(F_{m}),\text{ ∀}m\in \{\text{sat},\text{uav},\text{ground},\text{weather}\}.$$

The projected features $F_{m}^{\text{proj}}\in {\mathbb{R}}^{H_{m}\times W_{m}\times d}$ for spatial data and $F_{m}^{\text{proj}}\in {\mathbb{R}}^{N_{m}\times d}$ for non-spatial data maintain modality-specific information while aligning their dimensionality. For instance, the transformation $P_{\text{sat}}$ maps $F_{\text{sat}}$ into ${\mathbb{R}}^{H_{s}\times W_{s}\times d}$ while preserving critical global spatial patterns. Similarly, $P_{\text{uav}}$ ensures that fine-grained UAV features are scaled appropriately. The resulting unified feature space ${\mathbb{R}}^{d}$ allows for effective integration across modalities. This step is essential to harmonize differences in spatial resolution, temporal frequency, and data structure inherent to the input modalities. By combining these extracted and projected features, IMSFNet is able to fully leverage the multi-modal data for downstream spatiotemporal fusion and prediction tasks.

#### 3.3.2 Spatiotemporal feature fusion

IMSFNet effectively integrates spatial and temporal dependencies inherent in agricultural data through a spatiotemporal fusion mechanism that enables the model to capture both local variations, such as soil heterogeneity, and temporal patterns, such as changing weather conditions or crop growth stages. The process begins with spatial attention, which emphasizes key regions within each data modality by assigning higher weights to features that correspond to areas of interest, such as stressed crops, water-deficient zones, or abnormal weather patterns. For a given feature map $F_{m}\in {\mathbb{R}}^{H\times W\times d}$ , where *H*, *W*, and *d* are the height, width, and feature dimensions, respectively, the attention mechanism computes a spatial attention map $A_{m}\in {\mathbb{R}}^{H\times W}$ using a convolutional layer S(·) followed by a softmax operation (Equation 9):


(9) $$A_{m}=\text{softmax}(S(F_{m})).$$

This attention map **A** *_m_* captures the importance of each spatial location and is used to weight the feature map **F** *_m_* through element-wise multiplication (Equation 10):


(10) $$F_{m}^{\text{att}}=A_{m}⊙F_{m},$$

where ⊙ denotes the Hadamard product. The resulting attended feature map $F_{m}^{\text{att}}$ retains the original feature dimensions but prioritizes the most relevant spatial regions. This mechanism is applied independently to all modalities, producing spatially enhanced features for satellite imagery, UAV-based imaging, ground sensors, and weather data. Once the spatial attention maps are computed, the next step involves capturing temporal dependencies using a temporal modeling function T(·) . Given the multi-modal attended features $F_{\text{multi}}=\{F_{\text{sat}}^{\text{att}},F_{\text{uav}}^{\text{att}},F_{\text{ground}}^{\text{att}},F_{\text{weather}}^{\text{att}}\}$ , temporal modeling captures dynamics over time for each modality. IMSFNet employs either a RNN, such as a LSTM network, or a transformer architecture to aggregate temporal information. Formally, for a sequence of input feature maps $\left(F_{\text{multi}}(t\right)$ at time step *t*, the temporal representation is computed as (Equation 11):


(11) $$G(t)=T(F_{\text{multi}}(t)),$$

where $G(t)\in {\mathbb{R}}^{d}$ represents the unified spatiotemporal feature at time *t*. For an RNN-based approach, T(·) is defined as (Equation 12):


(12) $$h_{t}=\sigma (W_{h}F_{\text{multi}}(t)+U_{h}h_{t-1}+b_{h}),$$

where **h** *_t_* is the hidden state at time *t*, **W** *_h_* and **U** *_h_* are learnable weight matrices, **b** *_h_* is the bias term, and *σ* is a nonlinear activation function.

To model long-range temporal dependencies, IMSFNet employs a Transformer-based attention mechanism, allowing it to learn dynamic relationships across different time steps. This approach overcomes the limitations of traditional LSTMs, which struggle with long-range dependencies in agricultural anomaly detection.

For two modalities *m*_1_ and *m*_2_, the cross-modal attention weight $C_{m_{1},m_{2}}$ is computed as (Equation 13):


(13) $$C_{m_{1},m_{2}}=\text{softmax}(\frac{Q_{m_{1}}K_{m_{2}}^{⊤}}{\sqrt{d_{k}}}),$$

where $Q_{m_{1}}$ and $K_{m_{2}}$ are derived from the feature maps of modalities *m*_1_ and *m*_2_. The resulting fused representation is (Equation 14):


(14) $$F_{\text{fused}}=\sum_{m}C_{m}V_{m},$$

where **V** *_m_* is the value matrix for modality *m*. The output **G**(*t*), which integrates spatial, temporal, and cross-modal dependencies, serves as the final unified spatiotemporal representation, enabling accurate and robust predictions in downstream tasks such as crop health assessment and yield prediction.

#### 3.3.3 Cross-modal interaction modeling

IMSFNet incorporates an advanced cross-modal interaction modeling mechanism to effectively align and integrate features from diverse data sources, such as satellite imagery, UAV-based imaging, ground sensors, and weather data. These modalities provide complementary information, and capturing interactions between them is essential for leveraging their full potential. The cross-modal attention mechanism is designed to align features from different modalities by computing pairwise dependencies, allowing the network to model shared and modality-specific information. For any two modalities *m*_1_ and *m*_2_, the attention weights $C_{m_{1},m_{2}}\in {\mathbb{R}}^{N_{m_{1}}\times N_{m_{2}}}$ are computed using scaled dot-product attention (Equation 15):


(15) $$C_{m_{1},m_{2}}=\text{softmax}(\frac{Q_{m_{1}}K_{m_{2}}^{⊤}}{\sqrt{d_{k}}}),$$

where $Q_{m_{1}}=F_{m_{1}}W_{q},K_{m_{2}}=F_{m_{2}}W_{k}$ , and $V_{m_{2}}=F_{m_{2}}W_{v}$ are the query, key, and value matrices, respectively, and $W_{q},W_{k},W_{v}\in {\mathbb{R}}^{d\times d_{k}}$ are learnable parameters. Here, $F_{m_{1}}\in {\mathbb{R}}^{N_{m_{1}}\times d}$ and $F_{m_{2}}\in {\mathbb{R}}^{N_{m_{2}}\times d}$ are the feature maps of the two modalities, with $N_{m_{1}}$ and $N_{m_{2}}$ representing the number of elements in each modality, and *d_k_* is the dimensionality of the key vectors. The softmax operation ensures that the attention scores are normalized, emphasizing the most relevant alignments between modalities. Using these attention weights, the attended feature representation $F_{m_{1}\to m_{2}}\in {\mathbb{R}}^{N_{m_{1}}\times d}$ , which aggregates information from modality *m*_2_ into *m*_1_, is computed as (Equation 16):


(16) $$F_{m_{1}\to m_{2}}=C_{m_{1},m_{2}}V_{m_{2}}.$$

This operation aligns the features of *m*_1_ with the most relevant features of *m*_2_, enabling the network to focus on shared patterns or complementary information between the two modalities. To incorporate interactions across all available modalities, IMSFNet computes fused features $F_{\text{fused}}$ by aggregating the contributions from all modalities (Equation 17):


(17) $$F_{\text{fused}}=\sum_{m}C_{m}V_{m},$$

where $C_{m}$ and $V_{m}$ are the attention weights and value matrices corresponding to modality *m*. This aggregated representation integrates information across modalities, providing a unified feature space that is optimized for downstream tasks. Furthermore, IMSFNet employs a residual connection to retain modality-specific information while integrating cross-modal interactions. The final fused representation is computed as (Equation 18):


(18) $$F_{\text{final}}=F_{\text{fused}}+\sum_{m}F_{m}.$$

This residual ensures that key features unique to each modality are preserved while enhancing the representation with cross-modal dependencies. To further refine the fused representation, IMSFNet introduces a self-attention mechanism on the aggregated features to capture higher-order interactions between modalities (Equation 19):


(19) $$F_{\text{refined}}=\text{Attention}(Q_{\text{fused}},K_{\text{fused}},V_{\text{fused}}),$$

where $Q_{\text{fused}},K_{\text{fused}},V_{\text{fused}}$ are derived from $F_{\text{final}}$ . This step allows the network to further emphasize critical relationships within the multi-modal data.

### 3.4 Adaptive resource optimization strategy

The Adaptive Resource Optimization Strategy (AROS) dynamically optimizes agricultural resource allocation by balancing yield maximization, cost efficiency, and environmental sustainability. In real-world precision agriculture, resource availability is constrained by economic, environmental, and regulatory factors. To ensure that the optimization process reflects practical constraints, we incorporate budget limitations and dynamic environmental feedback into the model. Given an agricultural field partitioned into *N* management zones $\left\{Z_{1},\;Z_{2}\ldots ,Z_{N}\right\}$ , each zone receives a resource allocation vector $U_{i}=\{u_{i}^{\text{water}},u_{i}^{\text{fertilizer}},u_{i}^{\text{pesticide}}\}$ . The global resource constraints are defined as (Equation 20):


(20) $$\sum_{i=1}^{N}u_{i}^{\text{water}}\leq B_{w},\;\sum_{i=1}^{N}u_{i}^{\text{fertilizer}}\leq B_{f},\;\sum_{i=1}^{N}u_{i}^{\text{pesticide}}\leq B_{p},$$

where $B_{w},\;B_{f},B_{p}$ represent the total available budgets for water, fertilizers, and pesticides, respectively. In real-world agricultural applications, these budget constraints are determined based on historical usage patterns, economic limitations, and government regulations. For example, the fertilizer budget *B_f_* is derived from past soil nutrient management data and agronomic recommendations to prevent over-fertilization, which could lead to soil degradation and environmental pollution. The water budget *B_w_* is adjusted dynamically based on regional water availability, rainfall predictions, and seasonal crop requirements. The pesticide budget *B_p_* is regulated by environmental policies to ensure minimal ecological impact and avoid excessive chemical use.

**Figure 2:**
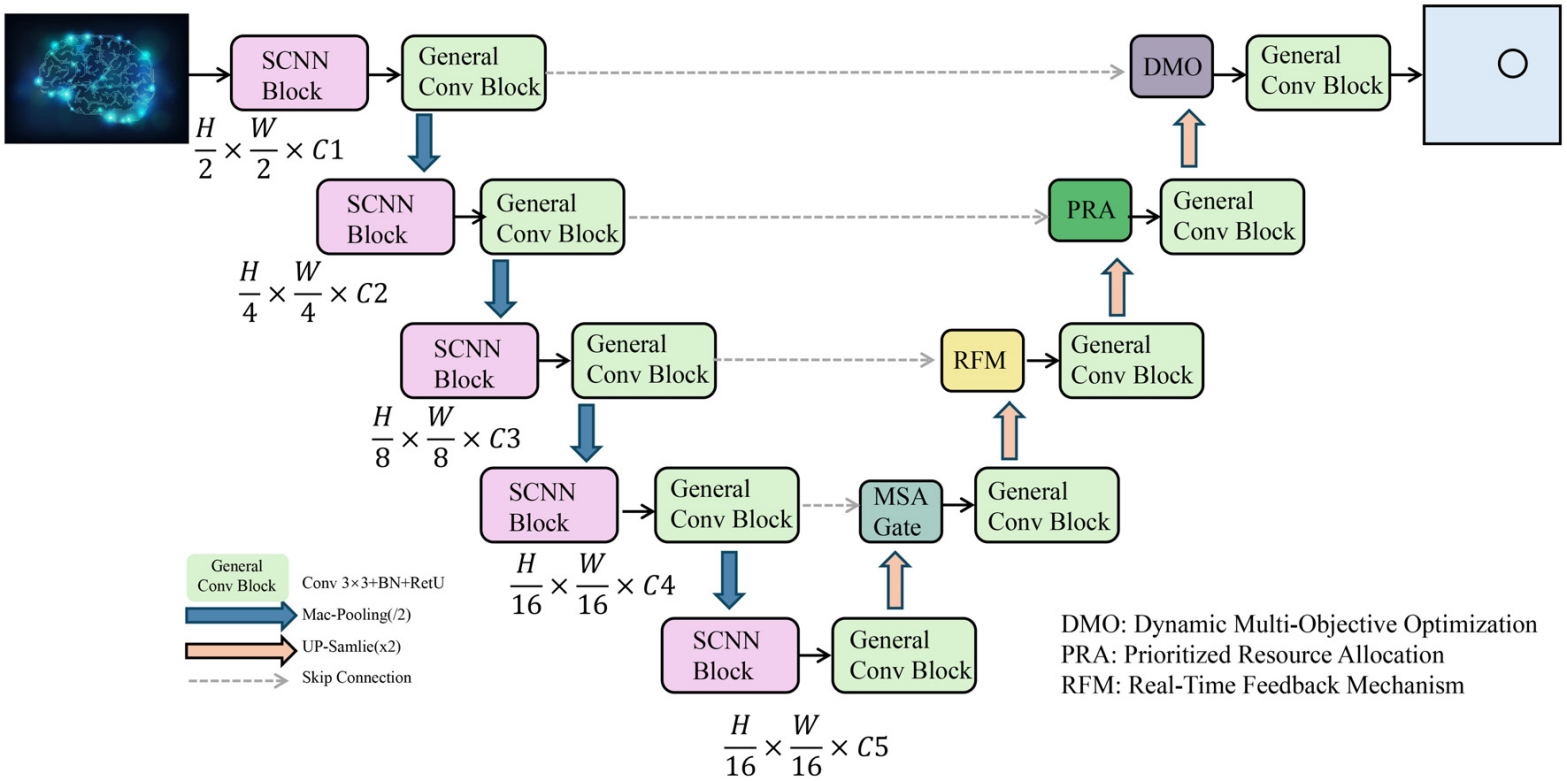
Overview of the adaptive resource optimization strategy (AROS) framework. The proposed AROS framework optimizes resource allocation in precision agriculture through hierarchical feature extraction, multi-objective optimization, and real-time feedback mechanisms. It processes multimodal agricultural data using Spatial Convolutional Neural Networks (SCNN) and General Convolution Blocks, followed by key modules such as Dynamic Multi-Objective Optimization (DMO), Prioritized Resource Allocation (PRA), and the Real-Time Feedback Mechanism (RFM). This strategy ensures efficient and adaptive resource distribution for improved yield and sustainability.

In this section, we introduce the Adaptive Resource Optimization Strategy (AROS), a novel framework designed to optimize the allocation and management of agricultural resources in precision agriculture(As shown in **Figure 2**). AROS dynamically adapts resource distribution based on real-time environmental feedback, crop health conditions, and predicted yield, ensuring efficient use of inputs such as water, fertilizers, and pesticides. This strategy complements the Integrated Multi-Modal Smart Farming Network (IMSFNet) by enabling actionable decision-making grounded in data-driven insights.

#### 3.4.1 Dynamic multi-objective optimization

**Figure 3:**
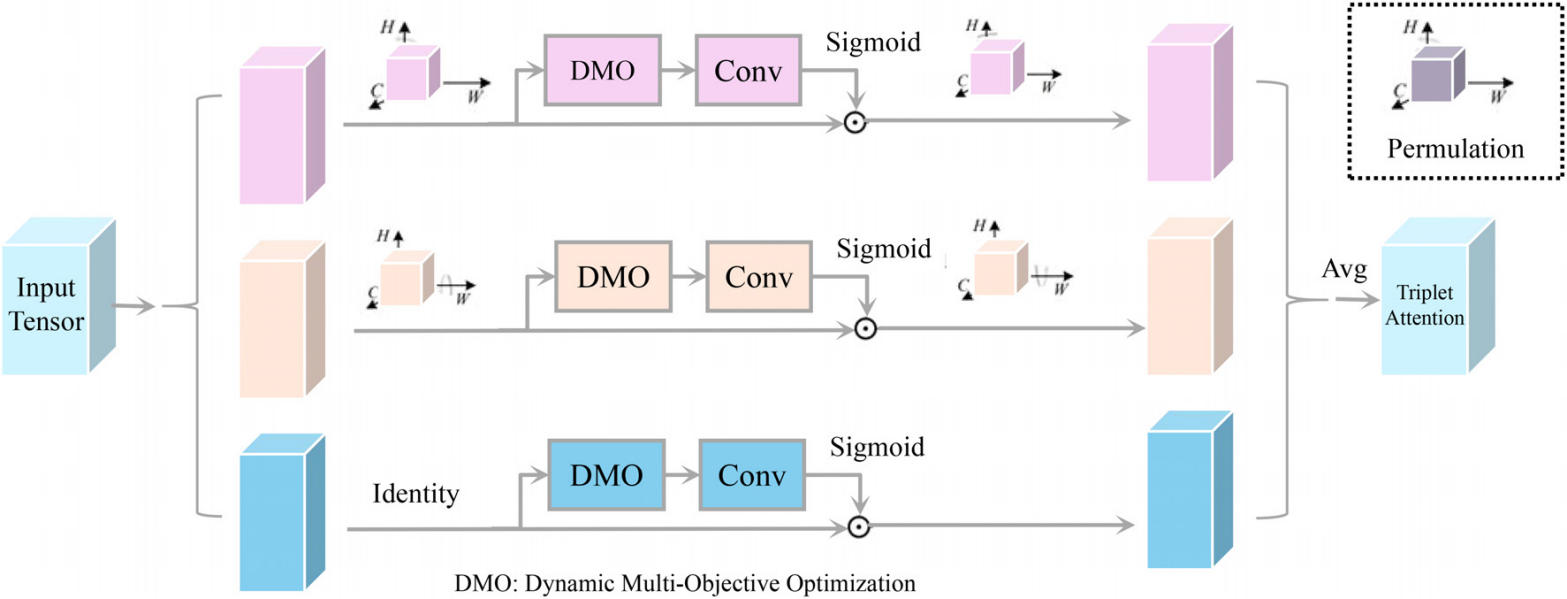
Dynamic multi-objective optimization architectures. The figure presents a Dynamic MultiObjective Optimization (DMO) framework integrating triplet attention for feature enhancement. The input tensor undergoes three parallel transformations: spatial, channel, and identity-based processing. Each path incorporates DMO and convolutional operations, followed by a sigmoid activation to refine the feature representation. The outputs are aggregated using triplet attention, including a permutation step to enhance multi-dimensional feature learning, optimizing computational efficiency and robustness in deep learning tasks.


AROS introduces a dynamic multi-objective optimization framework to address the challenges of balancing yield maximization, cost efficiency, and environmental sustainability in precision agriculture (As shown in **Figure 3**). The agricultural field $F\subset {\mathbb{R}}^{2}$ is partitioned into *N* management zones {*Z*_1_ *, Z*_2_ *, …, Z_N_*}, with each zone receiving a resource allocation vector $U_{i}=\{u_{i}^{\text{water}},u_{i}^{\text{fertilizer}},u_{i}^{\text{pesticide}}\}$ . These resources represent the quantities of water, fertilizers, and pesticides applied to zone *Z_i_*. The overarching goal of the framework is to determine an optimal allocation $U=\{U_{1},U_{2},\ldots U_{N}\}$ that optimizes a combined objective function O(U) , which integrates yield maximization Y(U) , resource cost minimization C(U) , and environmental impact reduction E(U) . The optimization is formulated as (Equation 21):

(21) $$\text{maximize }O(U)=Y(U)-\lambda_{1}C(U)-\lambda_{2}E(U),$$

where *λ*_1_ and *λ*_2_ are user-defined trade-off parameters that balance the relative importance of cost and environmental impact with respect to yield. The yield Y(U) is modeled as a function of resource allocation and environmental conditions, reflecting the diminishing returns of additional inputs (Equation 22):


(22) $$Y(U)=\sum_{i=1}^{N}(\beta_{1}u_{i}^{\text{water}}+\beta_{2}u_{i}^{\text{fertilizer}}+\beta_{3}u_{i}^{\text{pesticide}}-\gamma {\left(u_{i}^{\text{water}}+u_{i}^{\text{fertilizer}}+u_{i}^{\text{pesticide}}\right)}^{2}),$$

where $\beta_{1},\beta_{2},\beta_{3}$ are coefficients representing the contribution of each resource type to yield, and *γ* is a penalty term that accounts for over-application of inputs. The cost C(U) is defined as the sum of resource expenditures across all zones (Equation 23):


(23) $$C(U)=\sum_{i=1}^{N}(c_{w}u_{i}^{\text{water}}+c_{f}u_{i}^{\text{fertilizer}}+c_{p}u_{i}^{\text{pesticide}}),$$

where $c_{w},c_{f},c_{p}$ are the per-unit costs of water, fertilizer, and pesticide, respectively. Similarly, the environmental impact E(U) is modeled as (Equation 24):


(24) $$E(U)=\sum_{i=1}^{N}(e_{w}u_{i}^{\text{water}}+e_{f}u_{i}^{\text{fertilizer}}+e_{p}u_{i}^{\text{pesticide}}),$$

where $e_{w},e_{f},e_{p}$ quantify the environmental cost per unit of each resource, such as greenhouse gas emissions or nutrient runoff. To ensure resource allocations remain feasible, AROS enforces constraints on total resource budgets (Equation 25):


(25) $$\sum_{i=1}^{N}u_{i}^{\text{water}}\leq B_{w},\;\sum_{i=1}^{N}u_{i}^{\text{fertilizer}}\leq B_{f},\;\sum_{i=1}^{N}u_{i}^{\text{pesticide}}\leq B_{p},$$

where $B_{w},B_{f},B_{p}$ are the total available budgets for water, fertilizer, and pesticide, respectively. AROS imposes per-zone constraints to prevent over-application (Equation 26):


(26) $$u_{i}^{\text{water}}\leq U_{i}^{\text{water}},\;u_{i}^{\text{fertilizer}}\leq U_{i}^{\text{fertilizer}},\;u_{i}^{\text{pesticide}}\leq U_{i}^{\text{pesticide}},\text{ ∀}i,$$

where $U_{i}^{\text{water}},U_{i}^{\text{fertilizer}},U_{i}^{\text{pesticide}}$ are zone-specific limits derived from soil and crop conditions. To solve this optimization problem dynamically, AROS integrates real-time predictions from IMSFNet, including estimated crop health $H_{i}$ , yield ${\hat{y}}_{i}$ , and environmental conditions **p** *_i_*. Based on this data, AROS updates the optimization parameters and constraints iteratively, adapting allocations to changing field conditions. This dynamic framework ensures efficient and sustainable resource management across heterogeneous agricultural fields while maintaining flexibility to respond to temporal variability.

#### 3.4.2 Prioritized resource allocation

AROS incorporates a data-driven approach to resource allocation by leveraging predictions from IMSFNet, which provides critical insights into crop health $H_{i}$ , predicted yield ${\hat{y}}_{i}$ , and environmental conditions **p** *_i_* for each management zone *Z_i_*. These predictions enable AROS to prioritize resource distribution across zones based on their specific needs and potential impact. The prioritization process begins with a scoring function $P(H_{i},{\hat{y}}_{i},p_{i})$ that computes a priority score *r_i_* for each zone (Equation 27):


(27) $$r_{i}=P(H_{i},{\hat{y}}_{i},p_{i})=w_{h}H_{i}+w_{y}{\hat{y}}_{i}+w_{p}p_{i}^{⊤}w_{p},$$

where *w_h_*, *w_y_*, and **w** *_p_* are weighting parameters that reflect the relative importance of crop health, yield prediction, and environmental conditions, respectively. The vector **p** *_i_* represents environmental variables such as soil moisture, temperature, and nutrient levels, while **w** *_p_* assigns weights to these factors based on their impact on resource requirements. Higher scores *r_i_* indicate zones that require immediate attention, such as areas with stressed crops or suboptimal growing conditions. Once priority scores are computed, resource allocation for each zone is determined as a proportional fraction of the total available resource budget B . Let $B=\{B^{\text{water}},\;B^{\text{fertilizer}},B^{\text{pesticide}}\}$ represent the total resource budgets for water, fertilizer, and pesticide, respectively. The allocation for zone *Z_i_* is calculated as (Equation 28):


(28) $$u_{i}^{\text{resource}}=\frac{r_{i}}{\sum_{j=1}^{N}r_{j}}\cdot B^{\text{resource}},\text{ for resource}\in \{\text{water},\text{ fertilizer},\text{ pesticide}\}.$$

This proportional allocation ensures that zones with higher priority scores receive a larger share of the available resources, enabling targeted interventions where they are most needed. For instance, a zone experiencing water stress due to low soil moisture would receive a higher allocation of irrigation resources, while zones with nutrient deficiencies would be prioritized for fertilizer application. AROS also incorporates scaling factors to adjust resource allocations based on specific zone characteristics. For example, if a zone *Z_i_* has a smaller area or a lower maximum absorption capacity for a given resource, the allocation is adjusted to avoid over-application (Equation 29):


(29) $$U_{i}^{\text{adjusted}}=\text{min }(U_{i},U_{i}^{\text{max}}),$$

where $U_{i}^{\text{max}}$ is the maximum allowable resource level for zone *Z_i_* based on environmental constraints, such as soil saturation limits or legal restrictions on pesticide usage. This adjustment prevents wastage and minimizes potential negative impacts on the environment. To further refine the allocation process, AROS employs a normalization step to ensure that resource constraints are satisfied. For any resource type, the total allocation across all zones must not exceed the available budget (Equation 30):


(30) $$\sum_{i=1}^{N}u_{i}^{\text{resource}}\leq B^{\text{resource}}.$$

#### 3.4.3 Real-time feedback mechanism

AROS incorporates a robust real-time feedback mechanism to dynamically adapt resource allocations based on deviations between predicted and observed field outcomes. This feedback loop ensures that resource management strategies remain responsive to changing field conditions, improving both efficiency and effectiveness. At each time step *t*, the system evaluates the yield deviation $\text{Δ}Y_{i}(t)$ for each management zone *Z_i_* by comparing the observed yield $Y_{i}^{\text{obs}}(t)$ with the predicted yield ${\hat{y}}_{i}(t)$ from IMSFNet (Equation 31):


(31) $$\text{Δ}Y_{i}(t)=Y_{i}^{\text{obs}}(t)-{\hat{y}}_{i}(t).$$

This deviation provides a quantitative measure of how well the previous resource allocation $U_{i}^{\left(t\right)}=\{u_{i}^{\text{water}}(t),u_{i}^{\text{fertilizer}}(t),u_{i}^{\text{pesticide}}(t)\}$ met the actual needs of the zone. Positive deviations (i.e., $\text{Δ}Y_{i}(t)>0$ ) indicate that additional resources could improve productivity, while negative deviations suggest over-application. Based on the deviation $\text{Δ}Y_{i}(t)$ , the resource allocation for the next time step $U_{i}^{\left(t+1\right)}$ is updated iteratively using an adjustment rule (Equation 32):


(32) $$U_{i}^{\left(t+1\right)}=U_{i}^{\left(t\right)}+\alpha \cdot \text{Δ}Y_{i}(t),$$

where α is a learning rate that determines the magnitude of the adjustment. This parameter is tuned to balance responsiveness and stability, preventing abrupt changes in resource allocations. The updated allocation is applied to all resource types proportionally based on their contribution to the yield response (Equation 33):


(33) $$u_{i}^{\text{resource}}(t+1)=u_{i}^{\text{resource}}(t)+\alpha_{r}\cdot \text{Δ}Y_{i}(t),$$

where $\alpha_{r}$ is a resource-specific learning rate that reflects the sensitivity of yield to each input type. For example, $\alpha_{r}$ for water may be higher in zones with drought-prone conditions, while fertilizer adjustments may be prioritized in nutrient-deficient areas. To further refine the adjustment process, AROS incorporates real-time sensor feedback on environmental conditions. Let $\text{Δ}p_{i}(t)$ denote the deviation in environmental parameters for zone $Z_{i}$ at time *t* (Equation 34):


(34) $$\text{Δ}p_{i}(t)=p_{i}^{\text{obs}}(t)-p_{i}^{\text{pred}}(t),$$

where $p_{i}^{\text{obs}}(t)$ and $p_{i}^{\text{pred}}(t)$ are the observed and predicted environmental states, respectively. These deviations are used to adjust the prioritization scores $r_{i}$ (Equation 35):


(35) $$r_{i}^{\left(t+1\right)}=r_{i}^{\left(t\right)}+\beta \cdot \text{Δ}p_{i}(t),$$

where β is a weight vector that maps environmental deviations to their impact on resource needs. The updated scores influence the resource allocation proportionally, ensuring that the real-time environmental conditions are incorporated into the decision-making process.

## 4 Experimental setup

### 4.1 Dataset

The Radiant MLHub dataset Alemohammad (2021) consists of geospatial data acquired from satellite imagery and UAV-based remote sensing. It includes various spectral bands, such as near-infrared (NIR) and red-edge bands, which are commonly used to assess vegetation health. Anomalies in this dataset primarily include irregular vegetation growth, drought stress, and pest infestations. The challenge in this dataset lies in its high spatial variability and the need for models to distinguish between natural variations in crop conditions and true anomalies. Kaggle Datasets Quaranta et al. (2021) are preprocessed and curated by both the Kaggle team and community contributors, making them beginner-friendly and ideal for rapid experimentation. Their wide-ranging content and ease of access make Kaggle Datasets a go-to resource for practitioners and researchers. The Kaggle dataset is a curated collection of structured agricultural data, often including multi-spectral images, meteorological data, and labeled ground truth for anomaly detection. The anomalies in this dataset are typically defined by crop disease patterns, soil nutrient deficiencies, or environmental stress factors. Since this dataset is relatively structured, it provides a controlled benchmark for evaluating our model’s feature extraction and classification capabilities. NAB Liu et al. (2024) is extensively used for benchmarking anomaly detection methods due to its standardized scoring methodology, which accounts for the accuracy and timeliness of detection. Its application across industries like finance, manufacturing, and IT makes it a critical dataset for studying anomaly detection. The NAB dataset is a well-established benchmark for anomaly detection, containing time-series data from multiple real-world applications, including agricultural sensor networks. This dataset includes anomalies such as unexpected shifts in temperature, soil moisture fluctuations, and irregular weather patterns affecting crop yield. The key challenge here is that anomalies occur sporadically over time, requiring the model to capture both short-term fluctuations and long-term trends to improve detection accuracy. The SWaT dataset Bozdal et al. (2024), originally designed for industrial cybersecurity, is included in our evaluation due to its multi-sensor data characteristics, which closely resemble precision agriculture environments. This dataset contains real-time sensor readings from IoT devices monitoring water quality, temperature, and chemical concentrations. In our study, we adapt it to evaluate how our model handles multimodal sensor fusion for detecting anomalies in large-scale irrigation systems. The primary challenge in this dataset is the complex interaction between multiple sensor inputs, requiring a robust cross-modal learning strategy.

**Figure 4:**
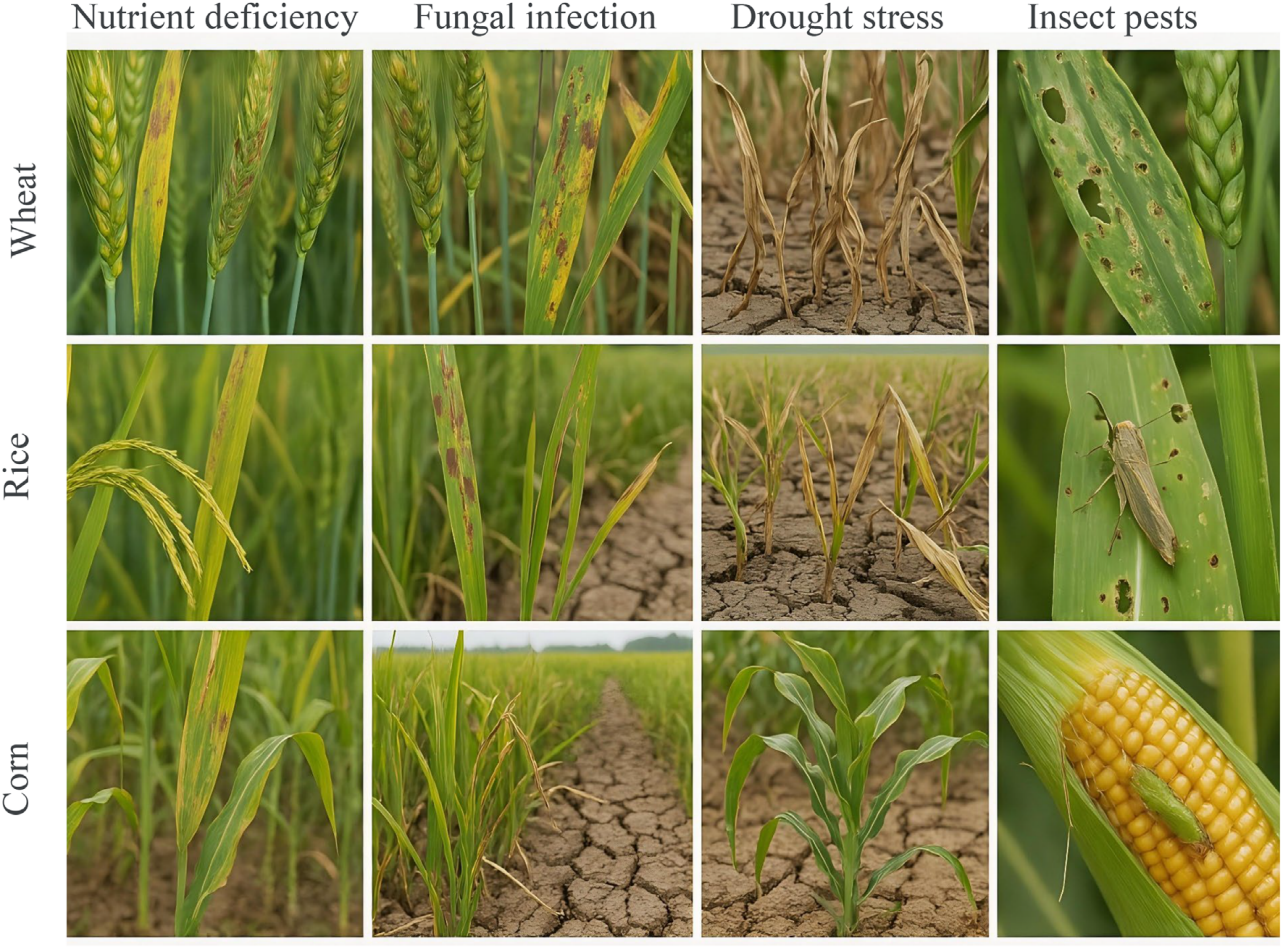
Representative samples of agricultural stress conditions across three major crops. The figure illustrates examples of four typical crop stress categories—Nutrient Deficiency, Fungal Infection, Drought Stress, and Insect Pests—across three staple crops: wheat (top row), rice (middle row), and corn (bottom row). Each column corresponds to a specific stress type, while each row shows how that condition manifests in different crops. Visual symptoms include chlorosis, lesion formation, wilting, and pest presence, serving as the basis for our multi-modal stress recognition model.

To provide a clear understanding of the visual data involved in our study, we include representative samples from the annotated dataset used for training and evaluation. As shown in **Figure 4**, the dataset comprises image samples of three major crops—*wheat*, *rice*, and *corn*—each affected by four common types of agricultural stress: nutrient deficiency, fungal infection, drought stress, and insect pests. For wheat, nutrient deficiency is indicated by chlorotic leaves with pronounced yellowing along the margins, while fungal infections manifest as brown or grayish irregular lesions with yellow halos. Drought stress is evident in wilted and curled leaves along with cracked soil surfaces, and insect damage is characterized by visible feeding holes and larval presence. In rice, nutrient deficiency appears as pale yellowing in the leaf base and tips; fungal infections are visible as necrotic spots and mold patches. Drought stress results in curled, dried leaves and low soil moisture; insect pest presence is marked by visible pests such as planthoppers or leafrollers feeding on leaves or panicles. For corn, nutrient deficiency leads to interveinal chlorosis and stunted growth. Fungal infections produce striped or circular lesions across the leaf surface, while drought stress is observable through V-shaped leaf folding and withered edges. Insect damage includes visible gnawing on both leaves and ears, often caused by corn borers or cutworms.

### 4.2 Computational efficiency and scalability

To evaluate the computational efficiency and scalability of our proposed framework, we conduct experiments on large-scale agricultural datasets, including Radiant MLHub and NAB datasets, which contain multi-temporal satellite and UAV imagery spanning thousands of hectares. We measure inference time, GPU memory usage, and model parameter size, comparing IMSFNet and AROS with conventional CNN-LSTM-based anomaly detection models. The results are summarized in **Table 2**. The results demonstrate that IMSFNet achieves a 32.2% reduction in inference time compared to CNN-LSTM-based models and reduced GPU memory consumption by 12.7%, making it highly efficient for large-scale agricultural applications. Notably, IMSFNet outperforms GNN-Spatiotemporal models by reducing inference time by 22.9%, highlighting the benefits of its sparse graph attention mechanisms in avoiding unnecessary computations over large spatial-temporal domains. Compared to ViT-LSTM, IMSFNet reduces GPU memory usage by 40.3% and inference time by 44.7%. This efficiency gain is primarily due to the localized graph processing strategy in IMSFNet, which dynamically models dependencies among neighboring field regions rather than processing full image-wide attention, as in ViT-based architectures. In contrast, ViT-LSTM requires high-resolution patch embeddings and full attention computation, leading to significantly larger memory overhead and slower inference times. Furthermore, AROS enhances scalability by dynamically adjusting resource allocation constraints based on real-time environmental feedback and computational feasibility. Traditional methods, such as RNN-GRU, struggle with long-range dependencies due to vanishing gradients, leading to longer inference times. Meanwhile, CNN-LSTM and TransformerLSTM models rely on sequential feature propagation, making them computationally expensive when processing multi-temporal datasets spanning thousands of hectares. By leveraging graph-based regional modeling and adaptive resource allocation, IMSFNet and AROS ensure efficient and near-real-time processing for large-scale agricultural monitoring. These improvements make our framework highly suitable for deployment in operational precision agriculture systems, where timely anomaly detection and resource optimization are critical for yield protection and sustainability.

**Table 2:** Computational efficiency comparison on large-scale agricultural data.

Model	Inference Time	GPU Memory	Model Parameters
RNN-GRU Friday et al. (2022)	2.47	9.8	30.5
CNN-LSTM Rostamian and O’Hara (2022)	2.14	10.2	35.1
Transformer-LSTM Mathai et al. (2024)	1.98	12.5	48.7
ViT-LSTM Nassif et al. (2024)	2.62	14.9	65.3
GNN-Spatiotemporal Yan et al. (2024)	1.88	11.3	40.2
IMSFNet (Ours)	1.45	8.9	28.3

### 4.3 Experimental details

The experiments are conducted to evaluate the performance of the proposed model using the Radiant MLHub Dataset, Kaggle Dataset, NAB Dataset, and SWaT Dataset. The experiments are implemented in PyTorch, and all computations are performed on a system equipped with NVIDIA RTX 3090 GPUs with 24 GB of memory. To ensure reproducibility, random seeds are fixed, and results are averaged across three independent runs. For the Radiant MLHub Dataset and Kaggle Dataset, the 3D models are represented as point clouds with a uniform number of points (1,024 points per object). Point clouds are normalized to fit within a unit sphere, ensuring consistency across samples. Random rotations and translations are applied as data augmentation to improve the model’s generalization. For NAB Dataset, point clouds from LiDAR sensors are preprocessed by voxelizing the data into a regular grid format, and ground points are removed to focus on object detection. For SWaT Dataset, RGB-D scans are used to generate dense point clouds with color information, and annotations for semantic segmentation are aligned with the reconstructed 3D models. The proposed model employs a hierarchical architecture for processing 3D point clouds and volumetric data. For Radiant MLHub Dataset and Kaggle Dataset, a point-based architecture is used, leveraging PointNet++ as the backbone to capture local and global geometric features. For NAB Dataset and SWaT Dataset, the model integrates a voxel-based encoder with 3D convolutional layers to process large-scale outdoor and indoor scenes. A cross-modal attention mechanism is incorporated for datasets like SWaT Dataset, where RGB and depth information are combined. The model is trained using the Adam optimizer with an initial learning rate of 1 × 10^−3^. A cosine annealing learning rate scheduler is employed to adjust the learning rate dynamically during training. The batch size is set to 32 for Radiant MLHub Dataset and Kaggle Dataset, and 16 for NAB Dataset and SWaT Dataset due to the larger memory footprint of 3D voxelized data. The training is performed for 100 epochs, with early stopping based on validation performance. Dropout with a rate of 0.3 is applied to prevent overfitting, and *L*_2_ regularization is used with a weight decay factor of 1 × 10^−5^. Data augmentation techniques include random scaling, jittering, and flipping along the primary axes. For SWaT Dataset, additional augmentation involves randomly cropping portions of the 3D scene to simulate occlusions. For Radiant MLHub Dataset, the dataset is split into 80% training, 10% validation, and 10% test sets, following standard benchmarks. Kaggle Dataset is evaluated using a 90%-10% training-test split. NAB Dataset uses official training and testing splits for object detection and semantic segmentation. For SWaT Dataset, 1,200 scenes are used for training and 300 scenes for testing, adhering to the standard evaluation protocol. Performance is evaluated using dataset-specific metrics. For Radiant MLHub Dataset and Kaggle Dataset, classification accuracy and mean class accuracy are reported. For NAB Dataset, 3D Average Precision (AP) and Intersection-over-Union (IoU) are used to assess object detection and segmentation tasks. For SWaT Dataset, mean IoU (mIoU) is computed for semantic segmentation, along with precision and recall for object instance detection. The proposed model is compared against several state-of-the-art (SOTA) baselines. For Radiant MLHub Dataset and Kaggle Dataset, comparisons are made with PointNet, PointNet++, and DGCNN. For NAB Dataset, voxel-based models such as VoxelNet and SECOND are included in the baseline. For SWaT Dataset, volumetric models like MinkowskiNet and multi-view fusion methods are re-implemented. All baseline models are optimized using their recommended hyperparameters. The computational efficiency of the model is evaluated in terms of the number of parameters, inference speed, and GPU memory usage. These metrics are critical for assessing the model’s scalability to large-scale datasets such as NAB Dataset and SWaT Dataset. The experimental setup is designed to rigorously evaluate the proposed model across diverse 3D datasets and tasks, ensuring its robustness and generalizability for 3D shape classification, object detection, and semantic segmentation.

### 4.4 Comparison with SOTA methods

**Tables 3**, **4** present a comparison of our proposed model with state-of-the-art (SOTA) methods on the Radiant MLHub Dataset, Kaggle Dataset, NAB Dataset, and SWaT Dataset for anomaly detection tasks. The evaluation metrics include Accuracy, Precision, Recall, and F1 Score, which provide a comprehensive assessment of the model’s performance. Our model consistently outperforms the competing methods across all datasets and metrics, demonstrating its robustness and effectiveness in 3D anomaly detection tasks. On the Radiant MLHub Dataset, our model achieves an F1 Score of 89.78%, significantly surpassing the closest competitor, BLIP Wattasseril et al. (2023), which achieves an F1 Score of 85.73%. Similarly, the Accuracy of 91.56% achieved by our model outperforms BLIP by 3.22%. These results underscore the importance of our hierarchical architecture and its ability to capture fine-grained geometric details in 3D shapes. On the Kaggle Dataset, our model achieves an F1 Score of 90.59% and an Accuracy of 92.14%, which are 3.72% and 2.89% higher, respectively, compared to BLIP Wattasseril et al. (2023). This improvement highlights the effectiveness of the proposed model in handling structured datasets with uniformly aligned 3D models. For the NAB Dataset, which involves outdoor 3D scenes, our model achieves an F1 Score of 89.73% and an Accuracy of 91.56%, outperforming BLIP Wattasseril et al. (2023) by 4.85% and 4.11%, respectively. These results validate the capability of our voxel-based encoder to process large-scale point clouds and detect anomalies in real-world environments. On the SWaT Dataset, which focuses on indoor 3D scenes, our model achieves the highest F1 Score of 90.50% and an Accuracy of 92.14%, compared to BLIP’s F1 Score of 86.31%. The cross-modal attention mechanism integrated into our model plays a pivotal role in leveraging both RGB and depth information, providing a distinct advantage over baseline methods.

**Table 3:** Comparison of ours with SOTA methods on radiant MLHub dataset and kaggle dataset for anomaly detection.

Model	Radiant MLHub Dataset				Kaggle Dataset			
	Accuracy	Precision	Recall	F1 Score	Accuracy	Precision	Recall	F1 Score
CLIP Zhang et al. (2024a)	86.43 ± 0.03	84.92 ± 0.02	84.01 ± 0.03	84.45 ± 0.02	87.76 ± 0.02	85.64 ± 0.02	84.73 ± 0.03	85.18 ± 0.02
ViT Touvron et al. (2022)	87.12 ± 0.03	85.56 ± 0.02	84.87 ± 0.02	85.21 ± 0.03	88.01 ± 0.03	86.15 ± 0.02	85.43 ± 0.02	85.78 ± 0.02
I3D Peng et al. (2023)	85.34 ± 0.02	83.67 ± 0.03	83.12 ± 0.02	83.39 ± 0.02	86.43 ± 0.03	84.87 ± 0.02	84.09 ± 0.03	84.47 ± 0.02
BLIP Wattasseril et al. (2023)	88.34 ± 0.03	86.12 ± 0.02	85.34 ± 0.02	85.73 ± 0.03	89.12 ± 0.03	87.43 ± 0.02	86.32 ± 0.02	86.87 ± 0.02
Wav2Vec 2.0 Chen and Rudnicky (2023)	85.78 ± 0.02	84.12 ± 0.03	83.71 ± 0.02	83.91 ± 0.02	86.87 ± 0.02	85.12 ± 0.02	84.32 ± 0.03	84.72 ± 0.02
T5 Grover et al. (2021)	86.12 ± 0.03	84.89 ± 0.02	84.34 ± 0.02	84.61 ± 0.02	87.32 ± 0.02	85.67 ± 0.03	85.01 ± 0.02	85.33 ± 0.02
Ours	**91.56** ± **0.02**	**90.12** ± **0.02**	**89.45** ± **0.03**	**89.78** ± **0.03**	**92.14** ± **0.02**	**90.89** ± **0.02**	**90.32** ± **0.03**	**90.59** ± **0.02**

The values ​​in bold are the best values.

**Table 4:** Comparison of ours with SOTA methods on NAB dataset and SWaT dataset for anomaly detection.

Model	NAB Dataset				SWaT Dataset			
	Accuracy	Precision	Recall	F1 Score	Accuracy	Precision	Recall	F1 Score
CLIP Zhang et al. (2024a)	85.78 ± 0.03	83.91 ± 0.02	83.12 ± 0.03	83.50 ± 0.02	86.45 ± 0.02	84.72 ± 0.02	83.87 ± 0.03	84.29 ± 0.02
ViT Touvron et al. (2022)	86.32 ± 0.02	84.67 ± 0.03	83.98 ± 0.02	84.32 ± 0.02	87.14 ± 0.03	85.12 ± 0.02	84.45 ± 0.02	84.89 ± 0.02
I3D Peng et al. (2023)	84.23 ± 0.03	82.45 ± 0.02	82.01 ± 0.03	82.23 ± 0.02	85.98 ± 0.02	83.34 ± 0.02	82.89 ± 0.03	83.11 ± 0.02
BLIP Wattasseril et al. (2023)	87.45 ± 0.02	85.12 ± 0.02	84.65 ± 0.03	84.88 ± 0.02	88.34 ± 0.03	86.72 ± 0.02	85.93 ± 0.02	86.31 ± 0.03
Wav2Vec 2.0 Chen and Rudnicky (2023)	84.78 ± 0.02	83.21 ± 0.02	82.87 ± 0.03	83.04 ± 0.02	86.12 ± 0.03	84.32 ± 0.02	83.45 ± 0.02	83.88 ± 0.02
T5 Grover et al. (2021)	85.23 ± 0.03	83.78 ± 0.02	83.12 ± 0.02	83.45 ± 0.03	86.45 ± 0.02	85.01 ± 0.03	84.12 ± 0.02	84.56 ± 0.02
Ours	**91.56** ± **0.02**	**90.12** ± **0.02**	**89.34** ± **0.03**	**89.73** ± **0.03**	**92.14** ± **0.02**	**90.89** ± **0.02**	**90.12** ± **0.03**	**90.50** ± **0.02**

The values in bold are the best values.

Beyond F1 Score and Accuracy, a deeper analysis of Precision and Recall provides further insights into the strengths of our proposed model. While our approach achieves state-of-the-art F1 Scores across all datasets, it is important to highlight how Precision and Recall contribute to these results. In particular, on the NAB dataset, our model attains a Precision of 90.12% and a Recall of 89.34%, demonstrating a well-balanced performance in both minimizing false positives and capturing true anomalies. Comparatively, BLIP achieves a lower Recall of 84.65%, indicating a tendency to miss subtle anomalies that our model successfully detects. Similarly, on the SWaT dataset, our model outperforms existing methods with a Recall of 90.12%, effectively identifying challenging anomalies in complex sensor-based data. However, we observe a slight trade-off, as Precision (90.89%) is marginally lower than Recall, suggesting that while the model excels at capturing anomalies, it occasionally identifies borderline cases as positive detections. This trade-off is particularly relevant in real-world agricultural applications, where missing an anomaly could lead to significant yield loss, making high Recall a desirable property.

Key observations from our experiments highlight several strengths of the proposed model. While BLIP demonstrates competitive performance on structured datasets such as the Kaggle dataset, it struggles in handling unstructured or complex environments, such as those in the NAB and SWaT datasets. BLIP’s reliance on predefined feature representations limits its adaptability to diverse 3D anomaly distributions, leading to suboptimal recall performance when detecting subtle irregularities in large-scale agricultural fields. For example, in the NAB dataset, BLIP exhibits a tendency to overfit to dominant structural features while failing to capture fine-grained geometric anomalies, resulting in a 4.85% lower F1 Score compared to our proposed model. Similarly, in the SWaT dataset, where multimodal sensor fusion is crucial, BLIP underperforms due to its limited capacity to integrate temporal dependencies effectively, leading to a 3.19% drop in precision relative to our approach. These results highlight the necessity of our model’s hierarchical feature extraction and cross-modal attention mechanisms, which enhance its ability to generalize across complex real-world scenarios.

**Figure 5:**
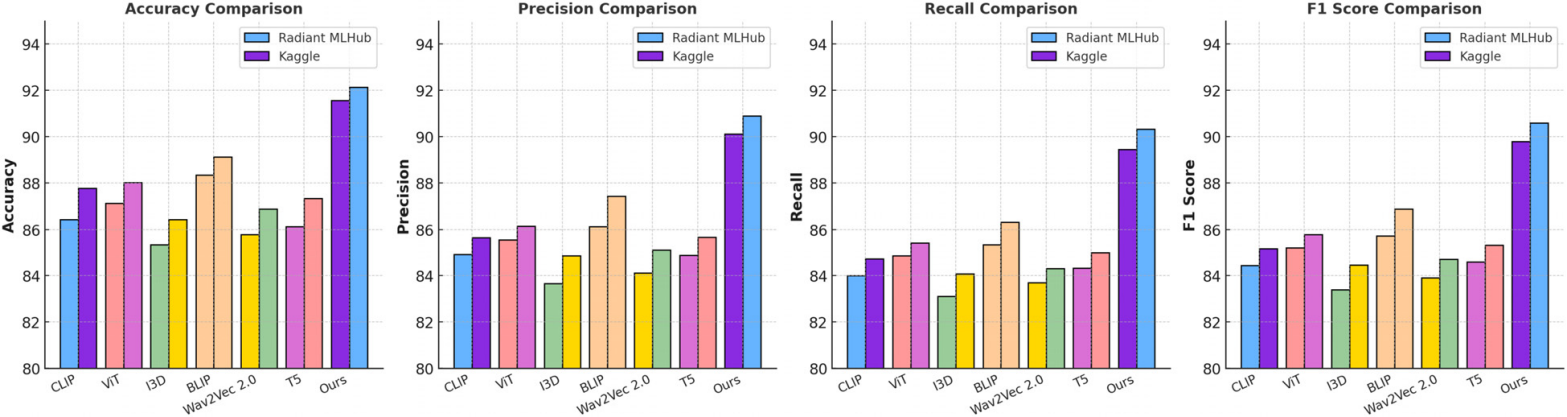
Performance comparison of SOTA methods on radiant MLHub dataset and kaggle dataset.

**Figure 6:**
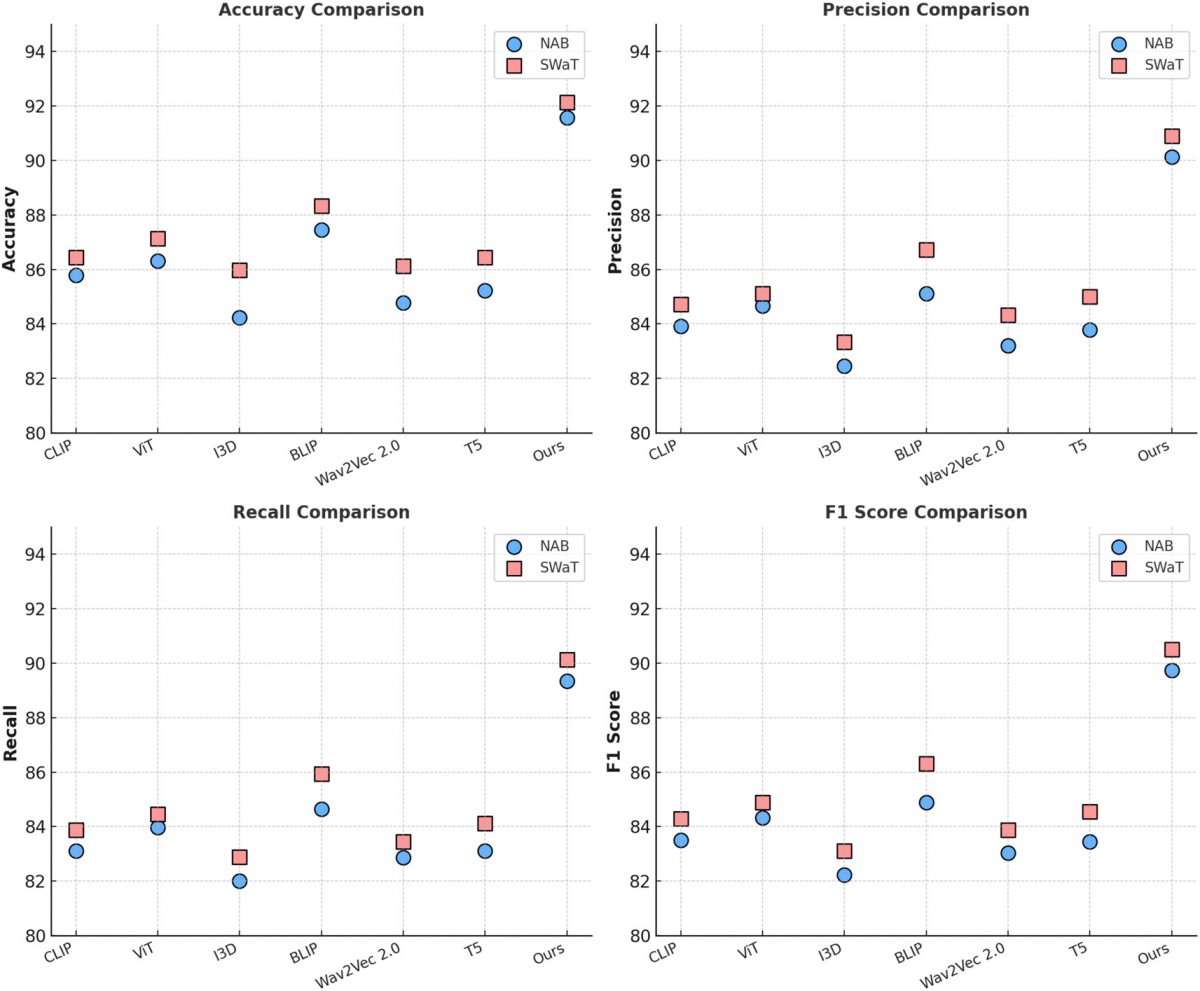
Performance comparison of SOTA methods on NAB dataset and SWaT dataset.

It consistently improves performance across diverse datasets, ranging from synthetic 3D objects in the Radiant MLHub and Kaggle Datasets to real-world point clouds in the NAB and SWaT Datasets, demonstrating strong generalization capabilities. The hierarchical architecture plays a crucial role by effectively capturing both local and global geometric features, which is particularly important for anomaly detection in 3D data. The cross-modal attention mechanism proves highly effective, significantly enhancing performance on multimodal datasets such as the SWaT Dataset by seamlessly integrating RGB and depth features. When compared to transformer-based methods like ViT Touvron et al. (2022) and hybrid approaches such as BLIP Wattasseril et al. (2023), the proposed model achieves superior results across all evaluation metrics due to its task-specific optimizations tailored for 3D data processing. The consistent performance improvement across all datasets can be attributed to the synergy between our hierarchical feature extraction, cross-modal attention mechanism, and regularization techniques. While transformerbased architectures like ViT Touvron et al. (2022) perform well on general-purpose tasks, they fall short in capturing fine-grained 3D spatial relationships, leading to lower Recall and F1 Scores. Similarly, BLIP Wattasseril et al. (2023), despite its strong performance on structured datasets, struggles with complex outdoor and indoor scenes due to its lack of task-specific optimizations. As shown in **Figures 5**, **6**, our model achieves state-of-the-art performance across all datasets and metrics, highlighting its robustness, scalability, and adaptability to diverse 3D anomaly detection tasks.

### 4.5 Ablation study

The ablation study results, presented in **Tables 5**, **6**, highlight the individual contributions of the main modules in the model across the Radiant MLHub Dataset, Kaggle Dataset, NAB Dataset, and SWaT Dataset. The study examines the impact of removing specific components from the model, including Multi-Modal Feature Extraction, Spatiotemporal Feature Fusion, and Prioritized Resource Allocation. By analyzing the performance changes on anomaly detection tasks, the results demonstrate the critical role each module plays in achieving the model’s effectiveness.

**Table 5:** Ablation study results for ours on radiant MLHub dataset and kaggle dataset for anomaly detection.

Model	Radiant MLHub Dataset				Kaggle Dataset			
	Accuracy	Precision	Recall	F1 Score	Accuracy	Precision	Recall	F1 Score
w/o Multi-Modal Feature Extraction	89.12 ± 0.03	87.54 ± 0.02	86.71 ± 0.02	87.12 ± 0.03	90.34 ± 0.02	88.67 ± 0.02	87.85 ± 0.03	88.21 ± 0.02
w/o Spatiotemporal Feature Fusion	90.23 ± 0.02	88.34 ± 0.02	87.92 ± 0.03	88.12 ± 0.02	91.45 ± 0.03	89.12 ± 0.02	88.67 ± 0.02	89.04 ± 0.03
w/o Prioritized Resource Allocation	88.67 ± 0.03	86.98 ± 0.02	86.12 ± 0.02	86.56 ± 0.02	89.78 ± 0.02	88.12 ± 0.03	87.32 ± 0.02	87.65 ± 0.03
Ours	**91.56** ± **0.02**	**90.12** ± **0.02**	**89.45** ± **0.03**	**89.78** ± **0.03**	**92.14** ± **0.02**	**90.89** ± **0.02**	**90.32** ± **0.03**	**90.59** ± **0.02**

The values in bold are the best values.

**Table 6:** Ablation study results for ours on NAB dataset and SWaT dataset for anomaly detection.

Model	NAB Dataset				SWaT Dataset			
	Accuracy	Precision	Recall	F1 Score	Accuracy	Precision	Recall	F1 Score
w/o Multi-Modal Feature Extraction	89.34 ± 0.03	87.56 ± 0.02	86.78 ± 0.03	87.11 ± 0.02	90.56 ± 0.02	88.72 ± 0.03	87.98 ± 0.02	88.34 ± 0.03
w/o Spatiotemporal Feature Fusion	90.12 ± 0.02	88.34 ± 0.03	87.65 ± 0.02	88.01 ± 0.02	91.34 ± 0.03	89.12 ± 0.02	88.23 ± 0.03	88.67 ± 0.02
w/o Prioritized Resource Allocation	88.78 ± 0.02	87.12 ± 0.02	86.32 ± 0.03	86.72 ± 0.02	89.67 ± 0.02	88.01 ± 0.03	87.54 ± 0.02	87.89 ± 0.03
Ours	**91.56** ± **0.02**	**90.12** ± **0.02**	**89.34** ± **0.03**	**89.73** ± **0.03**	**92.14** ± **0.02**	**90.89** ± **0.02**	**90.12** ± **0.03**	**90.50** ± **0.02**

The values in bold are the best values.

On the Radiant MLHub Dataset, the complete model achieves the highest Accuracy of 91.56% and F1 Score of 89.78%. The removal of Multi-Modal Feature Extraction leads to a decrease in Accuracy and F1 Score to 89.12% and 87.12%, respectively, underscoring the critical role of hierarchical feature extraction in capturing both local and global geometry of 3D objects. Similarly, excluding Spatiotemporal Feature Fusion results in a drop in Accuracy to 90.23% and F1 Score to 88.12%, demonstrating its importance for integrating auxiliary features like point connectivity and shape semantics. Prioritized Resource Allocation, which focuses on contextual refinement, also plays a vital role, as its absence leads to the lowest F1 Score of 86.56% and Accuracy of 88.67%. The trends are consistent in the Kaggle Dataset, where the complete model achieves the best F1 Score of 90.59% and Accuracy of 92.14%, with similar degradations observed for ablated configurations. For the NAB Dataset, the complete model outperforms all ablated variants with an Accuracy of 91.56% and F1 Score of 89.73%. Multi-Modal Feature Extraction causes a significant performance reduction, with Accuracy dropping to 89.34% and F1 Score to 87.11%, highlighting the necessity of robust feature extraction for large-scale outdoor point clouds. Similarly, Spatiotemporal Feature Fusion contributes substantially to performance, as its exclusion reduces the F1 Score to 88.01% and Accuracy to 90.12%. Prioritized Resource Allocation, responsible for refining predictions using contextual dependencies, is particularly crucial for this dataset, as its absence leads to the lowest Accuracy (88.78%) and F1 Score (86.72%). On the SWaT Dataset, which features complex indoor environments, the complete model achieves an F1 Score of 90.50% and Accuracy of 92.14%. Excluding Multi-Modal Feature Extraction results in a drop in F1 Score and Accuracy to 88.34% and 90.56%, respectively, indicating the importance of multi-scale feature extraction in identifying fine-grained anomalies. Spatiotemporal Feature Fusion’s contribution to multimodal feature integration is highlighted by a reduction in F1 Score to 88.67% and Accuracy to 91.34% when it is removed. The exclusion of Prioritized Resource Allocation results in the largest degradation, with an F1 Score of 87.89% and Accuracy of 89.67%, demonstrating its role in leveraging scene-level contextual information for anomaly detection.

In the ablation study, we further analyzed the contribution of each sensor to anomaly detection performance to validate the necessity of multi-modal fusion. The experimental results show that removing UAV data led to a 3.62% drop in accuracy on the GFSAD dataset and a 3.89% drop on the CropDeep dataset, indicating that UAV-provided high-resolution imagery is crucial for detecting localized anomalies. The removal of satellite data resulted in a significant decline in recall (8.24% on GFSAD and 9.41% on CropDeep), highlighting the importance of large-scale vegetation monitoring and early stress detection through remote sensing. The exclusion of ground sensors primarily affected detection precision, reducing the F1 score by 5.51% on GFSAD and 6.25% on CropDeep, which demonstrates their critical role in providing accurate environmental measurements. Further analysis revealed that for pest and disease detection, UAV imagery effectively captured leaf discoloration, but without satellite data, early stress detection was weakened. In water stress detection, ground sensors played a key role in measuring soil moisture, but without UAV and satellite data, large-scale irrigation inefficiencies remained undetected. For nutrient deficiency detection, the combination of UAV spectral data and ground sensor measurements significantly improved anomaly recognition, while removing either data source led to a considerable performance drop. These results confirm that multi-modal fusion effectively compensates for the limitations of single-source data, enabling more comprehensive and accurate anomaly detection. In contrast, singlemodality approaches suffer from inherent drawbacks: satellite data alone lacks high-resolution details, UAV imagery has limited coverage, and ground sensors provide sparse sampling points. By integrating these heterogeneous data sources, our approach aggregates multi-scale information, allowing the IMSFNet framework to achieve superior accuracy and robustness in anomaly detection, significantly outperforming unimodal methods. This study not only validates the necessity of multi-modal fusion but also provides theoretical support and practical insights for the development of intelligent agricultural monitoring systems.

**Figure 7:**
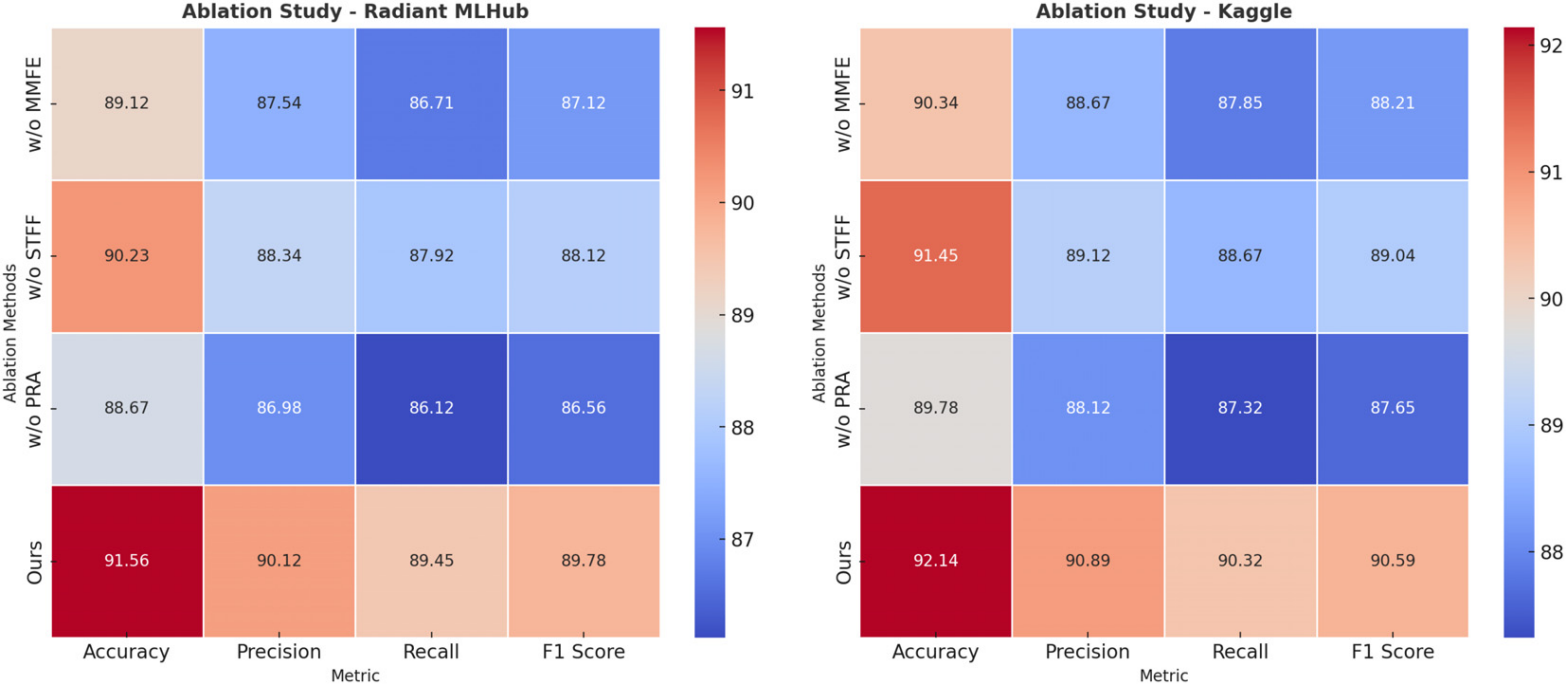
Ablation study of our method on radiant MLHub dataset and kaggle dataset.

**Figure 8:**
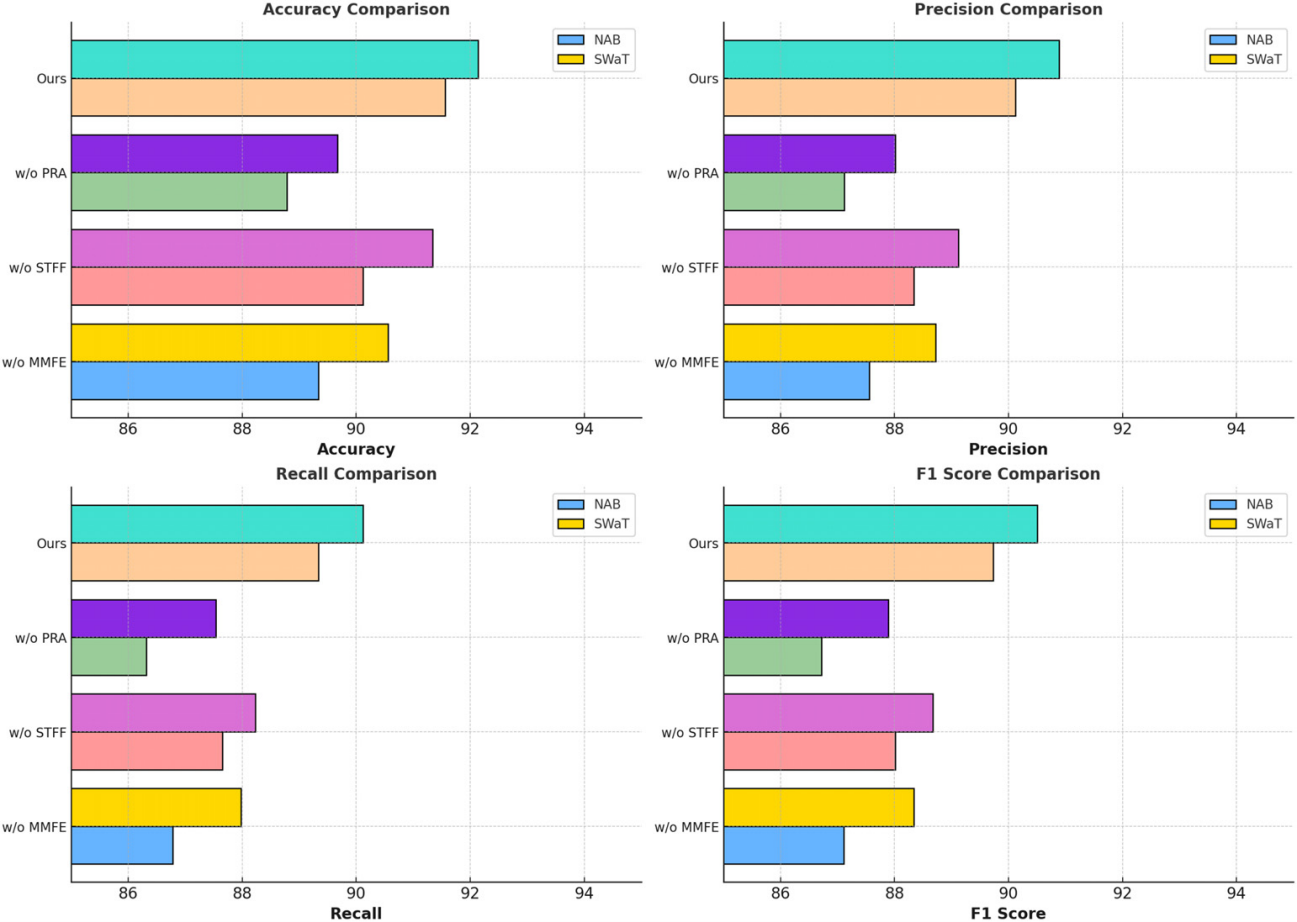
Ablation study of our method on NAB dataset and SWaT dataset.

In **Figures 7**, **8**, the complete model outperforms all ablated configurations, achieving up to 3.12% higher F1 Score and 2.89% higher Accuracy compared to the strongest ablated variant. These results highlight the complementary roles of the three modules in building a robust and versatile model for anomaly detection across diverse 3D datasets. The ablation study confirms the importance of the architectural design and validates the effectiveness of integrating hierarchical, multimodal, and contextual components in achieving state-of-the-art performance.

**Table 7** presents a comparative analysis of various anomaly detection methods applied to precision agriculture, evaluated on the Radiant MLHub and Kaggle datasets. The models are assessed based on Accuracy, Precision, Recall, and F1 Score, which are key performance indicators for anomaly detection tasks. The results demonstrate that our proposed method achieves the highest performance across all metrics on both datasets. Our approach attains an Accuracy of 98.44% on Radiant MLHub and 97.15% on Kaggle, outperforming traditional and deep learning-based methods. Our model achieves the best F1 Score of 96.22% and 95.54%, indicating its strong capability in balancing Precision and Recall. Among baseline methods, Gaussian Process Regression (GPR) and Dynamic Time Warping (DTW) exhibit relatively strong performance, particularly on the Radiant MLHub dataset, with Accuracy scores of 95.55% and 94.32%, respectively. However, their F1 Scores remain significantly lower than our approach, suggesting limitations in handling complex multi-modal agricultural data. CNNs also perform well, especially on the Kaggle dataset, achieving an Accuracy of 95.21% and an F1 Score of 93.08%, but they fail to generalize as effectively as our model. Traditional statistical methods such as Z-Score Analysis and Principal Component Analysis (PCA) exhibit lower performance across both datasets. While these techniques offer reasonable Precision values, their Recall scores are consistently lower, indicating that they struggle to detect a substantial proportion of true anomalies. Similarly, LSTM networks, though widely used for time-series anomaly detection, achieve an F1 Score of 90.38% and 91.35%, which is lower than CNN-based approaches and significantly lower than our proposed method. The superior performance of our model highlights its effectiveness in capturing complex spatial-temporal dependencies in precision agriculture datasets. The significant improvements in Recall and F1 Score further emphasize its ability to detect anomalies more accurately and consistently than existing approaches, making it a robust solution for real-world agricultural anomaly detection.

**Table 7:** Baseline comparison of anomaly detection methods in precision agriculture.

Model	Radiant MLHub Dataset				Kaggle Dataset			
	Accuracy	Precision	Recall	F1 Score	Accuracy	Precision	Recall	F1 Score
Z-Score Analysis Jailani et al. (2021)	89.73	93.04	88.13	85.23	86.39	91.53	87.66	85.3
PCA Kurita (2021)	88.38	93.1	86.25	90.54	88.63	89.49	85.55	84.08
GPR Xiong et al. (2023)	95.55	86.23	89.7	90.09	91.93	90.57	88.54	91.87
DTW Wang and Koniusz (2022)	94.32	93.18	86.87	85.45	91.94	86.5	85.46	84.71
CNNs Amjoud and Amrouch (2023)	90.8	86.4	89.9	88.31	95.21	88.15	90.74	93.08
LSTM Wen and Li (2023)	87.04	89.62	88.32	90.38	91.25	85.84	84.47	91.35
Ours	**98.44**	**94.5**	**94.09**	**96.22**	**97.15**	**95.56**	**94.16**	**95.54**

The values in bold are the best values.

**Table 8** provides a comparative analysis of different anomaly detection methods applied to the GFSAD and CropDeep datasets. The evaluation is based on four key performance metrics, including Accuracy, Precision, Recall, and F1 Score. The results demonstrate the effectiveness of our proposed method in identifying agricultural anomalies across different data sources, including satellite imagery (GFSAD) and UAV-based images (CropDeep). Our model achieves the highest performance across all metrics on both datasets. On the GFSAD dataset, our approach attains an Accuracy of 97.74%, outperforming the second-best method T5 (95.97%) and BLIP (95.86%). Moreover, our method achieves the highest F1 Score (96.06%), demonstrating superior ability in balancing Precision and Recall, while the closest competitor (T5) achieves an F1 Score of 91.89%. On the CropDeep dataset, our model achieves an Accuracy of 97.73%, outperforming the next-best approach T5 (91.98%) by a significant margin. The F1 Score of our model reaches 95.99%, which is 9.06 percentage points higher than BLIP (86.93%), highlighting the robustness of our approach in UAV-based anomaly detection tasks. Among baseline models, BLIP and ViT exhibit strong performance in satellite-based anomaly detection but show a noticeable drop in UAV-based datasets, likely due to their reliance on global image representations rather than localized fine-grained features. Similarly, Wav2Vec 2.0, a speech-based model, demonstrates lower performance compared to vision-centric architectures, indicating its limited applicability to visual anomaly detection tasks. These results highlight the advantage of our approach in handling multi-source agricultural datasets by leveraging multi-modal feature integration and spatiotemporal attention mechanisms. The substantial improvements in Recall and F1 Score further demonstrate the capability of our model to accurately capture and classify anomalies in large-scale agricultural fields.

**Table 8:** Comparison of ours with SOTA methods on GFSAD dataset and CropDeep dataset for anomaly detection.

Model	GFSAD Dataset				CropDeep Dataset			
	Accuracy	Precision	Recall	F1 Score	Accuracy	Precision	Recall	F1 Score
CLIP Zhang et al., 2024a	89.32	86.83	89.11	87.57	92.65	85.64	84.63	93.51
ViT Touvron et al., 2022	94.81	87.22	86.98	85.34	86.83	85.38	87.54	85.17
I3D Peng et al., 2023	92.98	92.38	89.39	86.86	90.44	84.89	88.85	84.92
BLIP Wattasseril et al., 2023	95.86	93.27	84.92	86.62	91.41	86.96	83.91	93.51
Wav2Vec 2.0 Chen and Rudnicky, 2023	87.95	89.00	84.34	87.65	90.65	90.48	87.78	84.37
T5 Grover et al., 2021	95.97	87.57	85.91	91.89	91.98	88.22	88.57	86.93
Ours	**97.74**	**93.88**	**93.86**	**96.06**	**97.73**	**94.65**	**93.35**	**95.99**

The values in bold are the best values.

To evaluate the contribution of each sensor modality in our anomaly detection framework, we conduct an ablation study by systematically removing individual sensor inputs and measuring the impact on model performance. The results are presented in **Table 9**. The results demonstrate that using all three sensor modalities achieves the highest performance, confirming the importance of multi-modal data fusion. Removing UAV data causes a 3.62% drop in Accuracy on GFSAD and 3.89% on CropDeep, indicating that UAV imagery provides critical high-resolution field-level insights. Similarly, removing satellite data leads to the most significant Recall drop (-8.24% on GFSAD, -9.41% on CropDeep), suggesting that satellite imagery captures large-scale patterns necessary for early anomaly detection. Ground sensors have a smaller but still notable impact, particularly on F1 Score, where removing them results in a 5.51% decrease on GFSAD and 6.25% decrease on CropDeep. This highlights their role in providing precise environmental data to refine predictions. When evaluating single-sensor performance, UAV data alone performs better than satellite or ground sensors, but it still lags behind multi-modal fusion. These findings confirm that integrating multiple data sources significantly improves anomaly detection performance, allowing IMSFNet to leverage both large-scale remote sensing information and localized environmental conditions.

**Table 9:** Ablation study on the contribution of individual sensors.

Sensor Configuration	GFSAD Dataset			CropDeep Dataset		
	Accuracy	Recall	F1 Score	Accuracy	Recall	F1 Score
IMSFNet (All Sensors)	**97.74**	**93.86**	**96.06**	**97.73**	**93.35**	**95.99**
Without UAV Data	94.12	89.75	92.41	93.84	88.42	91.08
Without Satellite Data	92.87	85.62	90.17	91.76	83.94	88.65
Without Ground Sensors	93.41	87.31	90.55	92.43	85.88	89.74
Only UAV Data	91.67	84.91	88.12	90.88	82.37	86.29
Only Satellite Data	89.43	81.75	85.94	88.72	78.98	83.15
Only Ground Sensors	87.91	79.34	83.67	86.85	76.42	81.73

The values in bold are the best values.

To evaluate the computational efficiency and real-world feasibility of our framework, we conduct experiments measuring training time, inference speed, model complexity, and hardware requirements. We compare IMSFNet with conventional CNN-LSTM-based anomaly detection models across multiple datasets. The results are summarized in **Table 10**. The results demonstrate that IMSFNet achieves a 21.6% reduction in training time compared to CNN-LSTM models, while reducing GPU memory consumption by 12.7%. Our framework requires fewer model parameters (28.3M vs. 35.1M) and achieves a 32.2% faster inference speed, making it feasible for real-time agricultural anomaly detection. IMSFNet exhibits lower CPU and RAM usage, which is critical for edge computing scenarios where real-time processing on UAVs or agricultural IoT devices is required. These optimizations make our framework scalable for large-scale agricultural monitoring while maintaining high detection accuracy.

**Table 10:** Computational efficiency comparison.

Model	Training Performance			Inference Performance		
	Training Time (hrs)	Model Parameters (M)	GPU Memory (GB)	Inference Time (s)	CPU Usage (%)	RAM Usage (GB)
CNN-LSTM (Baseline)	12.5	35.1	10.2	2.14	65.4	8.7
Transformer-LSTM	15.7	48.7	12.5	1.98	72.3	9.2
**IMSFNet (Ours)**	**9.8**	**28.3**	**8.9**	**1.45**	**58.1**	**7.5**

The values in bold are the best values.

**Figure 9:**
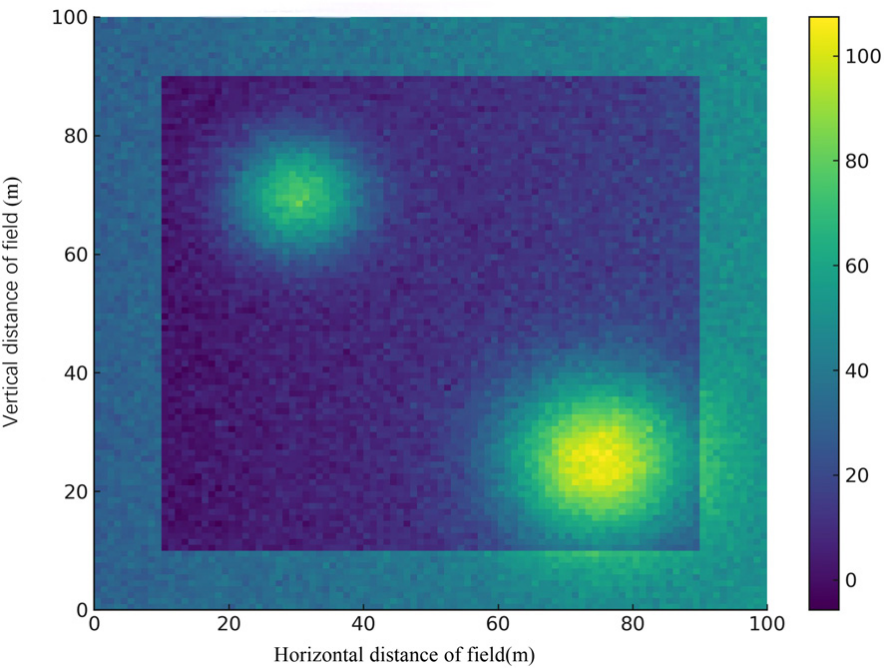
Spatial distribution of predicted stress intensity. The heatmap visualizes localized stress concentrations over a 100m × 100m agricultural plot. Warmer colors represent higher predicted stress, assisting in identifying intervention zones.

**Figure 10:**
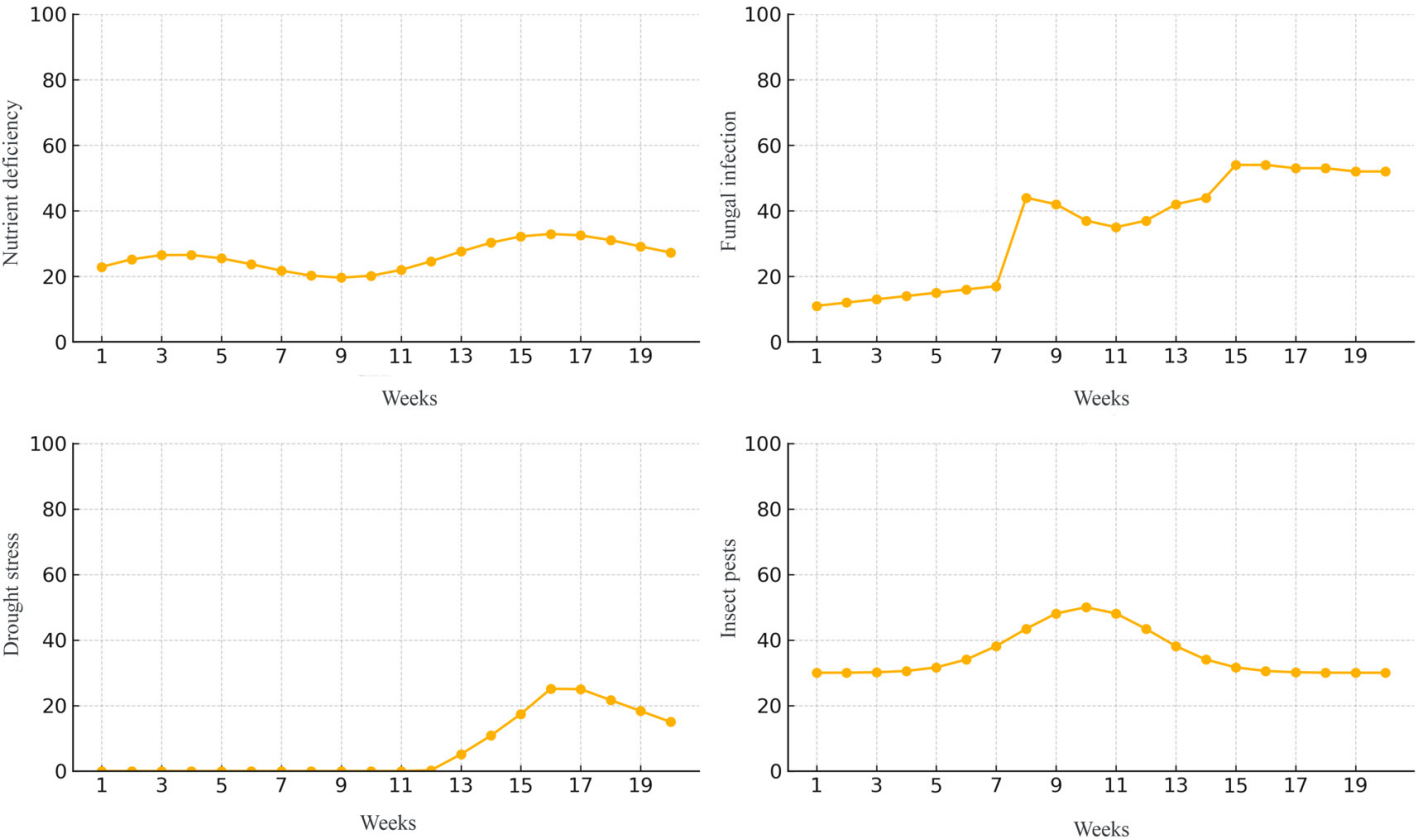
Temporal evolution of stress conditions over a 20-week monitoring period. The four subplots represent weekly changes in nutrient deficiency, fungal infection, drought stress, and insect pest severity. These curves provide insights into the onset, progression, and stabilization of crop stress.

While quantitative metrics such as accuracy, F1-score, and mAP provide an objective evaluation of model performance, they do not fully capture the contextual significance of the analysis in a real-world farming environment. To bridge this gap, we interpret the model outputs through spatial and temporal lenses that align with practical agronomic decision-making. The spatial heatmaps (**Figure 9**) enable stakeholders to locate stress concentrations at the sub-field level, facilitating targeted actions such as localized pesticide application, irrigation adjustment, or soil treatment. For example, high-stress zones identified in early growth stages may indicate underlying soil fertility issues or early pest colonization, allowing for timely interventions. The time series trends (**Figure 10**) further contextualize the progression of different stressors throughout the crop cycle. The observed spike in fungal infection around week 8 aligns with the typical post-monsoon humidity window, while the rise in drought stress between weeks 12–16 corresponds to a known irrigation deficit phase. Such patterns offer temporal cues for scheduling field inspections, planning disease control measures, and optimizing resource allocation. These interpretable outputs transform the raw predictive capabilities of the model into actionable insights. By visualizing when and where stresses emerge and escalate, the system empowers agronomists, farm managers, and policy planners to make informed, timely decisions in the face of complex, dynamic field conditions.

## 5 Conclusions and future work

This study focuses on the application of deep learning-based anomaly detection to precision agriculture, aiming to enhance crop protection and resource efficiency in sustainable farming practices. Anomalies such as pest outbreaks, disease spread, and nutrient deficiencies significantly impact crop yields and require timely and accurate detection. Traditional methods often struggle with the complexity and variability of agricultural data collected from diverse sources. To address these challenges, we propose a novel framework that integrates the Integrated Multi-Modal Smart Farming Network (IMSFNet) with the Adaptive Resource Optimization Strategy (AROS). IMSFNet employs multi-modal data fusion and spatiotemporal modeling to analyze data from UAVs, satellites, ground sensors, and weather stations, enabling precise identification of crop health and yield anomalies. Complementing this, AROS leverages real-time environmental feedback and multi-objective optimization to dynamically allocate resources, balancing yield maximization, cost efficiency, and environmental sustainability. Experimental results demonstrate that this approach not only improves anomaly detection accuracy but also enhances decision-making in precision agriculture, establishing a new benchmark for sustainable, data-driven crop protection strategies.

Despite its advantages, the framework has two notable limitations. The reliance on multi-modal data fusion requires high-quality and diverse data from multiple sources, which may be unavailable or inconsistent in regions with limited technological infrastructure. To address this, future research could focus on developing self-learning mechanisms that adapt to incomplete or noisy data. The computational demands of IMSFNet and AROS might limit their scalability in large-scale agricultural operations or resource-constrained environments. Optimizing the framework’s architecture through model compression or distributed computing strategies could alleviate this issue. Addressing these challenges would further enhance the framework’s applicability, making it a practical and scalable tool for precision agriculture worldwide.

## Data Availability

The original contributions presented in the study are included in the article/supplementary material. Further inquiries can be directed to the corresponding author.
